# Examining parent-of-origin effects on transcription and RNA methylation in mediating aggressive behavior in honey bees (*Apis mellifera*)

**DOI:** 10.1186/s12864-023-09411-4

**Published:** 2023-06-12

**Authors:** Sean T. Bresnahan, Ellen Lee, Lindsay Clark, Rong Ma, Michael Markey, Juliana Rangel, Christina M. Grozinger, Hongmei Li-Byarlay

**Affiliations:** 1 Huck Institutes of the Life Sciences, Pennsylvania State University, University Park, USA; 2 Agricultural Research and Development Program, Central State University, Wilberforce, USA; 3 Department of Biological Sciences, Wright State University, Dayton, USA; 4 HPCBio, University of Illinois at Urbana-Champaign, Champaign, USA; 5 Research Scientific Computing Group, Seattle Children’s Research Institute, Seattle, USA; 6 Department of Entomology, Texas A&M University, College Station, USA; 7 Department of Agricultural and Life Science, Central State University, Wilberforce, USA

**Keywords:** Honey bee, Intragenomic conflict, Transcriptomics, Oxford Nanopore direct RNA sequencing, Alternative splicing, Allele-biased transcription, RNA m6A

## Abstract

Conflict between genes inherited from the mother (matrigenes) and the father (patrigenes) is predicted to arise during social interactions among offspring if these genes are not evenly distributed among offspring genotypes. This intragenomic conflict drives parent-specific transcription patterns in offspring resulting from parent-specific epigenetic modifications. Previous tests of the kinship theory of intragenomic conflict in honey bees (*Apis mellifera*) provided evidence in support of theoretical predictions for variation in worker reproduction, which is associated with extreme variation in morphology and behavior. However, more subtle behaviors – such as aggression – have not been extensively studied. Additionally, the canonical epigenetic mark (DNA methylation) associated with parent-specific transcription in plant and mammalian model species does not appear to play the same role as in honey bees, and thus the molecular mechanisms underlying intragenomic conflict in this species is an open area of investigation. Here, we examined the role of intragenomic conflict in shaping aggression in honey bee workers through a reciprocal cross design and Oxford Nanopore direct RNA sequencing. We attempted to probe the underlying regulatory basis of this conflict through analyses of parent-specific RNA m6A and alternative splicing patterns. We report evidence that intragenomic conflict occurs in the context of honey bee aggression, with increased paternal and maternal allele-biased transcription in aggressive compared to non-aggressive bees, and higher paternal allele-biased transcription overall. However, we found no evidence to suggest that RNA m6A or alternative splicing mediate intragenomic conflict in this species.

**Supplementary Information:**

The online version contains supplementary material available at 10.1186/s12864-023-09411-4.

## Background

Conflict between genes inherited from the mother (matrigenes) and the father (patrigenes) is predicted to arise over parental investment in offspring or during social interactions among offspring, if these genes are not evenly distributed among offspring genotypes [1, 2]. According to the kinship theory of intragenomic conflict, matrigenes and patrigenes can each favor behaviors that promote their own respective fitness to the detriment of the other. This conflict drives the evolution of parent-specific transcription patterns in offspring that result from parent-specific epigenetic modifications [3]. Studies of intragenomic conflict and its epigenetic basis are therefore useful for revealing the molecular mechanisms that underlie the transgenerational inheritance of social behavior traits [4]. Given that matrigenes and patrigenes represent two transcriptional outcomes at individual genomic loci that can be assessed simultaneously within the same individual, studies of intragenomic conflict can also elucidate the regulation of gene expression more generally [5].

Honey bees (*Apis mellifera*) are an excellent model system in which to explore the mechanisms mediating intragenomic conflict, including parent-specific transcription and its epigenetic basis. The kinship theory of intragenomic conflict predicts that paternal alleles in polyandrous social insects will favor enhanced activity of reproductive traits [6]. For instance, honey bee workers typically increase their inclusive fitness by cooperating to support the queen’s reproductive fitness, even at the cost of their own personal reproductive fitness. However, under queenless conditions, cooperation between workers shifts to reproductive competition, whereby workers with developed ovaries aggress their sisters and lay unfertilized eggs that develop into haploid male drones [7–11]. Similarly, virgin (unmated) honey bee queens will fight to maintain their dominance by stinging other queens detected within the hive [12–15]. Thus, the alleles of genes underlying aggressive behaviors that enhance reproductive fitness may be in conflict, with paternal alleles favoring aggression and maternal alleles favoring cooperation.

Previous studies in honey bees have found evidence for parent-of-origin related variation in reproductive morphology, physiology, transcription [8, 16–22], and aggressive behavior [14, 23], all of which are consistent with the kinship theory of intragenomic conflict [6, 24]. These studies evaluated workers from reciprocal crosses of “Africanized” honey bees (AHBs, which are derived from the subspecies *A. m. scutellata*) and European honey bees (EHBs, which are derived from subspecies from Europe, such as *A. m. ligustica*) – two strains of honey bee that vary in their genotype, physiology, and behavior [25, 26]. The activation and size of ovaries in reciprocal crosses of AHBs and EHBs is greater in workers with AHB paternity as compared to EHB paternity, and this phenotypic difference is associated with enrichment for paternal allele-biased transcription in the ovaries, fat bodies [21], and brains [22]. Moreover, studies of worker aggression in hybrid crosses of AHBs and EHBs suggest that aggressive behaviors are increased by AHB paternity [14, 27–29]. Therefore, in this study, we tested the hypothesis that aggression (specifically focusing on aggressive stinging behavior in defense of the colony) is increased in soldier-aged workers with AHB paternity relative to workers with EHB paternity, and that this pattern is associated with an enrichment for paternal allele-biased transcription.

For most of the genes exhibiting parent-specific transcription in plants and mammals, differential DNA methylation of promoters or associated noncoding regulatory regions underlies their parent-of-origin transcription [30, 31]. Consistent with predictions of the kinship theory of intragenomic conflict [6, 24], previous studies have demonstrated that parent-specific transcription occurs in multiple tissues and developmental stages in social insects, including honey bees [17, 19, 21, 22] and bumble bees [32]. However, these transcriptional patterns are not associated with allelic DNA methylation differences in either species [32, 33]. This is perhaps unsurprising, given that differential DNA methylation does not appear to be associated with transcriptional variation in social insects specifically [34], or insects generally [35]. Thus, how parent-specific transcription is regulated in social insects remains an open area of investigation [36].

RNA methylation has recently been recognized as an important gene regulatory mechanism [37–39]. N6-methyladenosine (m6A) is the most abundant eukaryotic RNA modification, accounting for more than 80% of all RNA methylation [40]. In mammals, it plays a role in neurogenesis and morphology [41] and alters synaptic transmission by regulating transcript abundance of protein coding genes involved in neuronal signaling [42]. m6A affects RNA structure, splicing, localization, translation, stability, and metabolism [43, 44] and thus, allele-specific m6A modifications may drive differences in allele-specific transcript abundance [45]. In honey bees, recent studies revealed that chemical suppression of m6A impacts larval development and caste determination [46] and that fat body and brain tissues in workers show variation in global levels of m6A [47]. Whether there is a relationship between m6A and behavior in honey bees has yet to be investigated, however. Here, we used Oxford Nanopore direct RNA sequencing to assess (1) the relationship between parent-specific m6A and parent-specific transcription and (2) the association between each of these mechanisms with parent-of-origin effects on an aggressive behavior in soldier-aged worker honey bees from reciprocal crosses of AHBs and EHBs. We tested the hypothesis that parent-specific m6A underlies parent-specific transcription, with higher transcript abundance of the unmethylated allele.

Additional layers of gene regulation – including alternative splicing and the activities of long intergenic non-coding RNAs (lincRNAs) – have also revealed insights into the molecular basis of honey bee social behavior [48–51]. Work in other insect and mammalian model species have revealed roles for lincRNAs in development, disease, behavior, and metabolism [52–55]. With few exceptions [56], most studies of alternative splicing and long noncoding RNAs in honey bees have used short-read sequencing or cDNA microarrays [57, 58], which are limited to detecting only full-length isoforms or alternative splicing events [59, 60]. Here, we utilized long-read sequence data to profile full-length non-coding RNA molecules to study their relevance to parent-of-origin effects on honey bee aggression. We described the alternative splicing and lincRNA profiles of soldier-aged worker honey bees, compared between aggressive and non-aggressive individuals, and assessed the relationships between alternative splicing, parent-specific m6A, and parent-specific transcript abundance. We tested the specific hypothesis that genes which show parent-specific m6A or transcript abundance also show alternative splicing.

## Results

### Parent-of-origin effects on honey bee worker aggression

In contrast with previous work [14, 27–29], we observed inconsistent parent-of-origin effects on worker aggression across our four experimental colonies (Table 1). Colony 1 showed significantly higher aggression in workers with EHB paternity than those with AHB paternity, whereas Colony 2 (from which we isolated RNA from the head and thorax of individual bees for sequencing) and Colony 3 showed higher aggression in workers with AHB paternity. In Colony 4, aggression was slightly higher in workers with AHB paternity, but this difference was not statistically significant. When data from all colonies were combined, there was no statistically significant parent-of-origin effect on aggression.

**Table 1:** Frequency of aggressive stinging observed in workers from reciprocal crosses of EHBs and AHBs.

Block	Colony		% of individuals with EHB mother observed stinging		% of individuals with AHB mother observed stinging		χ^2^ test *p-*value
A	1		12.62%		0.00%		$3.77\cdot {10}^{-13}$
	2		2.43%		7.89%		$0.0098$
B	3		1.11%		15.77%		$2.03\cdot {10}^{-8}$
	4		4.39%		5.00%		$0.8324$
Combined			Average % of individuals with EHB mother observed stinging	SD	Average % of individuals with AHB mother observed stinging	SD	Logistic regression *p-*value
			5.17%	5.14%	7.17%	6.60%	0.8685

### Parent-specific allele-biased transcription associated with worker aggression

At approximately 60x genome coverage, we detected an average of 1.24 million homozygous single nucleotide polymorphisms (SNPs) per diploid queen and 2.29 million SNPs per haploid drone. We identified a total of 31,078 unique transcripts in our samples, including 3,640 lincRNAs. Of these transcripts, 3,081 (9.91%) were shared between both crosses and sufficiently varied in their sequence identity between the parents of each cross, allowing for identification of parent-of-origin reads in the workers. Specifically, we identified 35,782 SNP positions within transcripts that had at least 2 SNPs. Of the 3,081 transcripts, 2,584 were from the published annotation (NCBI *Apis mellifera* Annotation Release 104), and 497 were novel. After filtering SNP positions with low read counts in the workers, our dataset contained read counts at 18,674 positions distributed among 1,928 transcripts (approximately 9.69 SNPs per transcript) in non-aggressive workers and 33,494 positions distributed among 2,820 transcripts (approximately 11.88 SNPs per transcript) in aggressive workers.

**Fig. 1:**
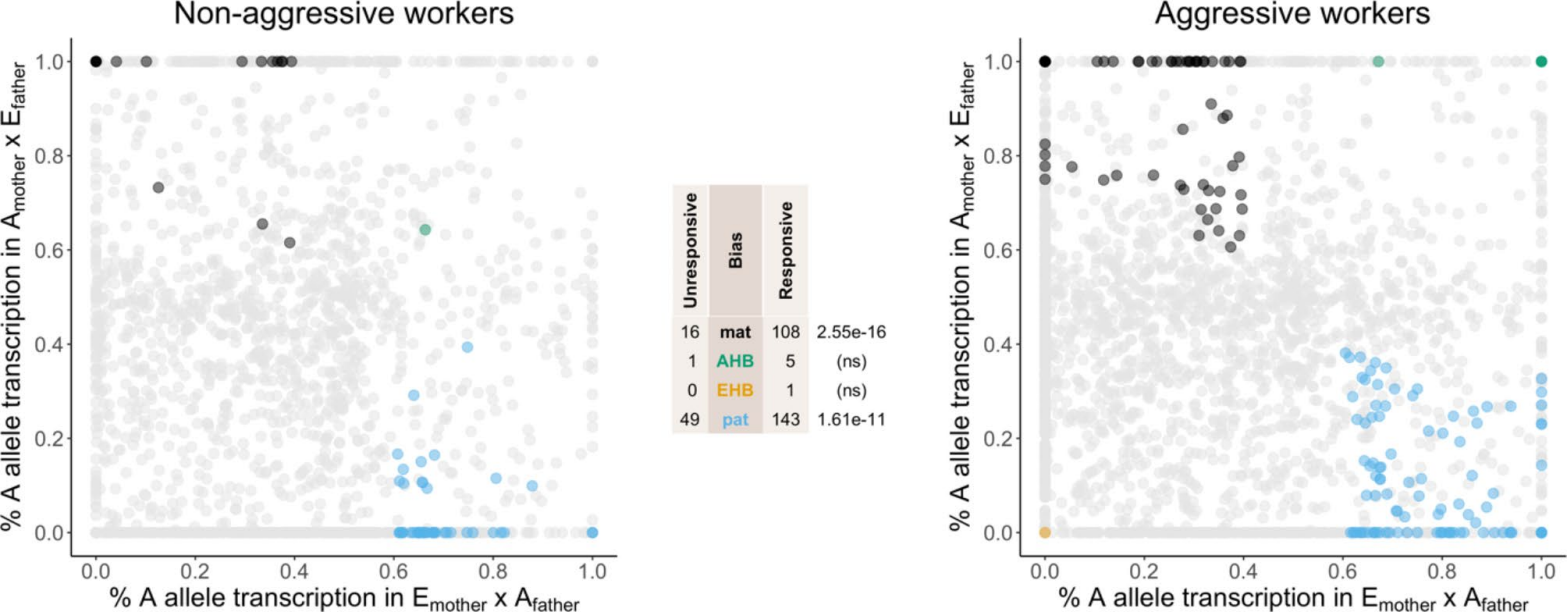
Worker aggression is associated with increased maternal and paternal allele-biased transcription. Transcript abundance of parent and lineage alleles were assessed in non-aggressive and aggressive workers from an EHB and AHB reciprocal cross. The x-axis represents, for each transcript (1,928 in non-aggressive workers and 2,820 in aggressive workers), the average proportion of AHB reads in workers with an EHB mother and AHB father (*p1*), and the y-axis represents, for each transcript, the average proportion of AHB reads in workers with an AHB mother and EHB father (*p2*). Each color represents a transcript which is significantly biased at all tested SNP positions: black is maternal, green is AHB, gold is EHB, blue is paternal, and gray is not significant. Significance was determined using the overlap between two statistical tests: a generalized linear interactive mixed model (GLIMMIX) [17, 21, 22], and a Storer-Kim test along with previously established cutoff thresholds [61] of *p1* < 0.4 and *p2* > 0.6 for maternal bias, *p1* > 0.6 and *p2* < 0.4 for paternal bias, *p1* < 0.4 and *p2* < 0.4 for EHB bias, and *p1* > 0.6 and *p2* > 0.6 for AHB bias

We identified 312 transcripts that showed allelic bias in both crosses (Fig. 1), including 231 from previously annotated genes, 68 novel transcripts, and 13 lincRNAs. Some transcripts showed allele-biased transcription in both non-aggressive and aggressive workers (*n* = 11), whereas other transcripts showed allelic bias in only one group (non-aggressive only, *n* = 55; aggressive only, *n* = 246). In total, in non-aggressive workers, 66/1,928 (3.42%) of tested transcripts showed allelic bias, whereas in aggressive workers, 256/2,820 (9.111%) of tested transcripts showed allelic bias. See Supplementary Dataset 1 (Table S16) for a complete listing of the parent and lineage biased transcripts identified in this study. In support of our hypothesis, we found that paternal allele-biased transcripts were enriched in aggressive workers (χ^2^*p* = 1.61 * 10^− 11^). Interestingly, maternal allele-biased transcripts were also enriched in aggressive workers (χ^2^*p* = 2.55 * 10^− 16^), although there were fewer maternal allele-biased transcripts overall. Cytoplasm cellular compartment (GO:0005737) was overrepresented for paternal allele-biased transcripts in aggressive workers. No biological process terms or KEGG pathways were associated with paternal allele-biased transcripts in non-aggressive workers, or with maternal allele-biased transcripts in either non-aggressive or aggressive workers.

We found little overlap between the genes showing allele-biased transcription associated with aggression in our study and (1) genes identified in a previous study [23] as showing parent-of-origin expression in guard bees, and (2) genes within quantitative trait loci (Sting 1–3) associated with aggressive behaviors in honey bees [62]. Specifically, we identified four genes showing allele-biased transcription in both our study and Gibson et al., only two of which were biased toward the same allele (Supplementary Dataset 1, Table S18). No overlap was found between genes showing allele-biased transcription in our study and genes within the Sting 1–3 QTLs (Supplementary Dataset 1, Table S19).

### Parent-specific RNA m6A associated with worker aggression

**Fig. 2:**
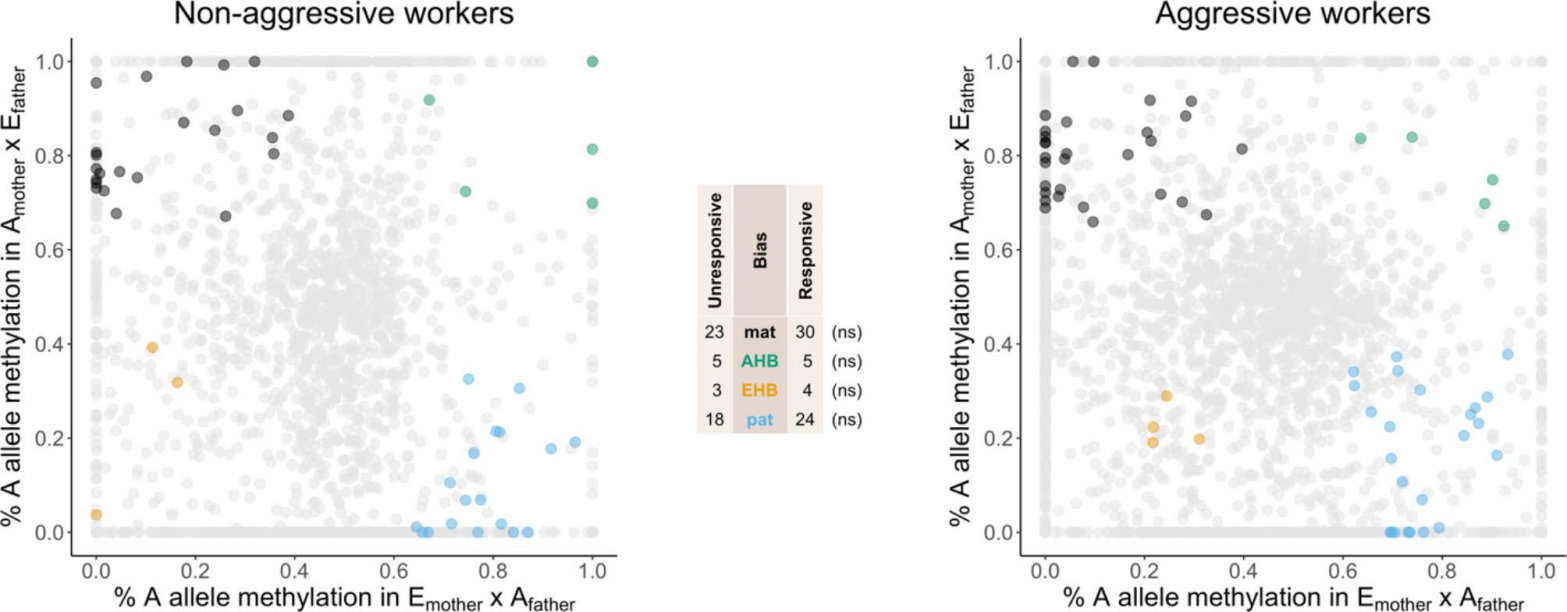
Worker aggression is not associated with an increase in parent or lineage specific RNA m6A. Read abundance and m6A rate of parent and lineage alleles were assessed in non-aggressive and aggressive workers from an EHB and AHB reciprocal cross. The x-axis represents, for each transcript (1,928 in non-aggressive workers and 2,820 in aggressive workers), the average proportion of AHB reads that were methylated in workers with a EHB mother and AHB father, whereas the y-axis represents, for each transcript, the proportion of AHB reads that were methylated in workers with an AHB mother and EHB father. Each color represents a transcript which is significantly biased at all tested SNP positions: black is maternal, green is AHB, gold is EHB, blue is paternal, and gray is not significant. Significance was determined using an unpooled two-tailed *z*-test [63] along with previously established cutoff thresholds [61] of *p1* < 0.4 and *p2* > 0.6 for maternal bias, *p1* > 0.6 and *p2* < 0.4 for paternal bias, *p1* < 0.4 and *p2* < 0.4 for EHB bias, and *p1* > 0.6 and *p2* > 0.6 for AHB bias

We identified 103 transcripts that showed parent or lineage biases in RNA m6A in both crosses (Fig. 2), including 87 from previously annotated genes, 15 novel transcripts, and 1 lincRNA. Some transcripts showed allele-biased m6A in both non-aggressive and aggressive workers (*n* = 9), whereas others showed allelic bias in only one group (non-aggressive only, *n* = 40; aggressive only, *n* = 54). See Supplementary Dataset 1 (Table S17) for a complete listing of the transcripts with parent and lineage biases in m6A identified in this study. In contrast to transcription, there was no relationship between parent-specific m6A and worker aggression. Additionally, there were no gene ontology terms overrepresented for genes showing paternal nor maternal allele-biased m6A.

Only three genes which showed allele-biased transcription also showed allele-biased m6A, each of which showed bias toward the paternal allele in both transcript abundance and m6A levels. In aggressive workers, this included one of two transcript isoforms for elongation of very long chain fatty acids protein AAEL008004 (LOC724552; GB54396). In non-aggressive workers, this included the transcript for an uncharacterized gene (LOC726321) and one of four transcript isoforms for vitellogenin (LOC406088; GB13999; GB49544).

### Differential expression

We detected transcript abundance of 19,092 genes and 3,640 lincRNAs in our samples. After filtering low-count genes, our dataset consisted of transcript levels of 14,462 genes and 2,415 lincRNAs. Of these, 8,058 (55.72%) of the genes and 451 (18.67%) of the lincRNAs were from the published annotation. When the crosses were analyzed separately, we identified one significantly differentially expressed gene (DEG) at a false discovery rate (FDR) < 0.05 between the non-aggressive and aggressive bees in each cross: box A-binding factor (LOC725389; GB50932) in cross A, and myrosinase 1 (LOC411978; GB54486) in cross B. However, a full model accounting for differences between crosses revealed that there were no significant DEGs between these behavioral phenotypes in our study.

### Detection of lincRNAs and isoform switching

In total, we detected 2,415 lincRNAs from all samples, 11 of which were differentially expressed (see Supplementary Dataset 1, Table S3). However, none of these showed parent-specific transcription or m6A biases. Additionally, we detected few genes that were significant for alternative splicing or isoform switching (see Supplementary Dataset 1, Table S2). Five genes were detected which had significant isoform switches (FDR < 0.1) between non-aggressive and aggressive bees, with putative functional consequences: synaptic vesicle glycoprotein 2B (LOC409924; GB41065), adenylosuccinate synthetase (LOC409299; GB14705; GB43704), H(+)/Cl(-) exchange transporter 7 (LOC413069; GB51847), protein YIF1B (LOC551459; GB48886), and hypoxia up-regulated protein 1 (LOC551763; GB54222). None of these showed significant parent-specific transcription or m6A biases. Models of the genes exhibiting splicing and isoform switching are available in Supplementary Dataset 2 (Figures S21-S44).

## Discussion

In eusocial insects like *A. mellifera*, the alleles of genes underlying aggressive behaviors that enhance reproductive fitness are predicted to be in conflict, with paternal alleles favoring aggression, and maternal alleles favoring cooperation. Here, we tested the hypothesis that an aggressive behavior (i.e., stinging) in honey bee workers should exhibit parent-of-origin effects and should be associated with enriched paternal allele-biased transcription. Additionally, we examined whether certain gene regulatory mechanisms play a role in intragenomic conflict. Indeed, we found that both paternal and maternal alleles showed significant increases in transcription for different genes in aggressive versus non-aggressive individuals. However, there was no evidence for parent-of-origin effects on RNA m6A or alternative splicing. Moreover, there was no association between genes that showed parent-specific transcription, m6A, or alternative splicing (see Supplementary Dataset 1).

In four reciprocally crossed colonies of “Africanized” and European honey bees (AHBs and EHBs, respectively), we tested the hypothesis that aggression should be increased in workers with AHB paternity relative to workers with EHB paternity. While we observed significant parent-of-origin effects on this behavior in three of the four colonies, the results were inconsistent between them, with only colonies 2 and 3 showing significantly more aggression in workers with AHB paternity, and colony 1 not showing any stinging behavior in workers with AHB paternity. While our study contrasts with previous reports of overall higher aggression in workers with AHB paternity [14, 27–29], those studies also reported behavioral variation between colonies. Additionally, we used a mtDNA haplotype analysis [64] to identify F0 AHB queens collected from feral colonies and confirm the maternal lineage of our managed F0 EHB queens. While we can be certain that the mtDNA haplotypes of these F0 queens were indeed AHB and EHB, respectively, it is possible that paternal alleles from the reciprocal lineage could have been present elsewhere in their genomes. Moreover, Rittschof et al. [65] demonstrated that honey bee workers which experienced higher levels of aggression within their colonies during pre-adult stages of development showed increased aggression in adulthood relative to siblings which experienced lower levels of aggression. We did not assess aggression in workers of the parental colonies and so did not control for this in our study. Therefore, our study was limited in that we were unable to disentangle the extent to which the higher level of aggression observed in workers with AHB paternity was due to inherited factors or environmental differences experienced during pre-adult stages of these bees.

Using a colony that exhibited significant differences in aggression associated with paternity, we next explored whether there were parent-specific differences in transcription associated with increased aggression. In contrast with our hypothesis that aggression should be associated with an increase in paternal allele-biased transcription, we found a significant increase in both paternal and maternal allele-biased transcription in aggressive bees relative to non-aggressive bees, although there were more paternal allele-biased than maternal allele-biased transcripts in aggressive bees. This concomitant increase in maternal allele-biased transcription could be explained by between-locus conflicts, whereby some genes have been selected to be preferentially expressed from the maternal allele to counteract the effects of genes that have been selected to be preferentially expressed from the paternal allele. This is supported by the observation in plants and mammals that imprinted genes (those that exhibit parent-specific transcription driven by parent-specific epigenetic marks) are frequently co-expressed [66]. In this context, it is hypothesized that within-locus conflicts, which are resolved by *cis*-regulatory mechanisms like DNA methylation and repressive histone marks [31], select for secondary conflicts between loci [67]. Consequently, extensions to *trans*-regulatory interactions between imprinted genes have been shown to follow naturally from the kinship theory of intragenomic conflict in both theoretical and empirical studies of imprinted gene networks [66]. However, we did not detect an enrichment for any similar gene ontology terms or KEGG pathways among the genes showing parent-specific transcription in our study.

Transgenerational epigenetic inheritance of RNA m6A has been described in brine shrimp (*Artemia franciscana*) [68] and has been hypothesized to modulate offspring metabolism through paternal environmental exposures which affect spermatogenesis [69]. Therefore, we hypothesized that m6A may underlie parent-specific transcription patterns in offspring. To our knowledge, our study is the first analysis to examine the relationship between parent-specific RNA m6A and parent-specific transcription in a behavioral context. We tested the hypothesis that parent-specific m6A underlies parent-specific transcription, with the unmethylated allele being expressed, and the methylated allele being silenced. While we identified many transcripts that exhibited parent- and lineage-biased m6A in aggressive bees, few also showed allele-biased transcription, none which matched our expectation of higher transcript abundance of the unmethylated allele. We also examined whether parent-specific transcription is associated with alternative splicing. When examining the transcript profiles of aggressive and non-aggressive bees, we identified a handful of genes that showed differential splicing patterns. Some of the differentially spliced genes had functions that could play a role in behavioral variation, including neuronal function and stress response. There was, however, no overlap between genes that showed differential splicing and genes that showed parent-specific transcription.

We did not identify any significant DEGs between aggressive and non-aggressive bees in our study. This was unexpected, as several previous studies had identified many DEGs associated with aggression in honey bees [25, 70–74], but not surprising given that our sequencing libraries were constructed from RNA pooled from multiple transcriptionally distinct tissues [75] and had lower read depth than what is typically used for statistical tests of differential expression. Additionally, genes that showed alternative splicing or isoform switching tended to be highly expressed, which suggests that our ability to identify genes with splice variants was related to our read depth. The recommended input for direct RNA sequencing reactions is 500 ng of mRNA (Direct RNA Sequencing Kit SQK-RNA002, Oxford Nanopore Technologies, Oxford, UK), and we were only able to isolate approximately 15 µg total RNA per individual bee from which approximately 150 ng mRNA was captured. Thus, the relatively low read depth in our study was due to low input to the library preparations. Moreover, our method assessed colony-level aggression, which may not clearly reflect the aggressiveness state of individual bees. We attempted to control for behavioral differences attributable to age (all bees in our colonies were age-matched) or differences in exposure to the stimuli (we disrupted each colony with noise, physical agitation, and the introduction of alarm pheromone), but individual behavior states may not be adequately described by one piece of qualitative information (stung / did not sting) recorded during one ten-minute observation period. Future studies could instead record the unstimulated response of individual bees towards an intruder in an arena assay at multiple timepoints, as described in [71], which allows for measuring quantitative information (latency for attack).

The molecular mechanisms underlying parent-specific transcription in insects remain unidentified [36], and some understanding of these mechanisms is necessary to direct investigations into the nature of their establishment and inheritance. In plants and mammals, imprinted genes, which exhibit parent-specific transcription driven by parent-specific epigenetic marks [5], are canonically regulated by DNA methylation of promoter or enhancer regions [31] that is, at least in mammals, established in primordial germ cells and remains present after fertilization [76]. In honey bees, queens mate with multiple drones [77], and evidence suggests that DNA methylation profiles of worker bees are more similar within than between patrilines [78]. However, DNA methylation is not associated with parent-specific transcription in honey bees [33]. Recently, maternally inherited histone 3 (H3) methylation was demonstrated to drive the expression of “non-canonically” imprinted genes, which do not exhibit parent-specific differences in DNA methylation [31, 79, 80]. Variation in H3 methylation has been associated with transcriptional variation and caste differentiation during honey bee larval development [81], and evidence suggests that the honey bee genome is partitioned into regulatory domains with differential enrichment for H3 methylation with respect to worker ovarian plasticity [82]. Thus, future studies should examine the role of chromatin and histone post-translational modifications in mediating intragenomic conflict in honey bees.

The lack of correlation between genes that show parent-specific transcript abundance and significant differences in overall transcript abundance between behavioral phenotypes in honey bees [21, 22] is puzzling. Future studies should examine the potential regulatory linkages between these genes to determine if subtle changes in transcript abundance caused by parent-specific transcription can cascade to larger changes in transcript abundance of genes found in the same network. For example, Galbraith et al. (2021) found that genes showing differences in parent-specific transcript abundance in worker bee brains were overrepresented in gene co-expression network modules associated with metabolism, nutrition, certain cell signaling pathways (i.e., FOXO, MAPK, and HIPPO), and spliceosomes [22]. Targeted studies of the functional consequences of parent-specific transcription of individual genes in these networks would be highly informative. It is also possible that these observed biases in parent-specific transcript abundance are isolated to specific cell types [83], and our study was limited in that we sequenced material from pools of two transcriptionally distinct tissues (heads and abdomens) [75]. Therefore, future studies should utilize single tissues and/or single cell sequencing technologies for finer resolution [84, 85]. Finally, we sequenced tissue from a total of only 12 individuals (3 aggressive and 3 non-aggressive from each of the reciprocal cross pairs), which limited our power to detect transcriptional and regulatory variation. Therefore, future studies should be performed with a higher sample size, if possible.

## Conclusions

This study provides evidence for intragenomic conflict in worker honey bee aggression. When coupled with previous studies that demonstrated how intragenomic conflict shapes worker reproduction [17, 19, 21, 22], our results suggest that intragenomic conflict and parent-specific transcription may be important factors in shaping the individual variation seen within the context of social behavior in honey bees, and potentially other species [24, 32]. We examined whether RNA m6A and/or alternative splicing play a role in intragenomic conflict in honey bees and did not find evidence to suggest that either are associated with parent-specific transcription in this species. Thus, the molecular mechanisms underlying parent-specific transcription in honey bees remain unidentified [33], and insights from recent studies of genomic imprinting in plants and mammals [31, 79–82] provide direction for future investigations.

## Methods

### Biological samples

**Fig. 3:**
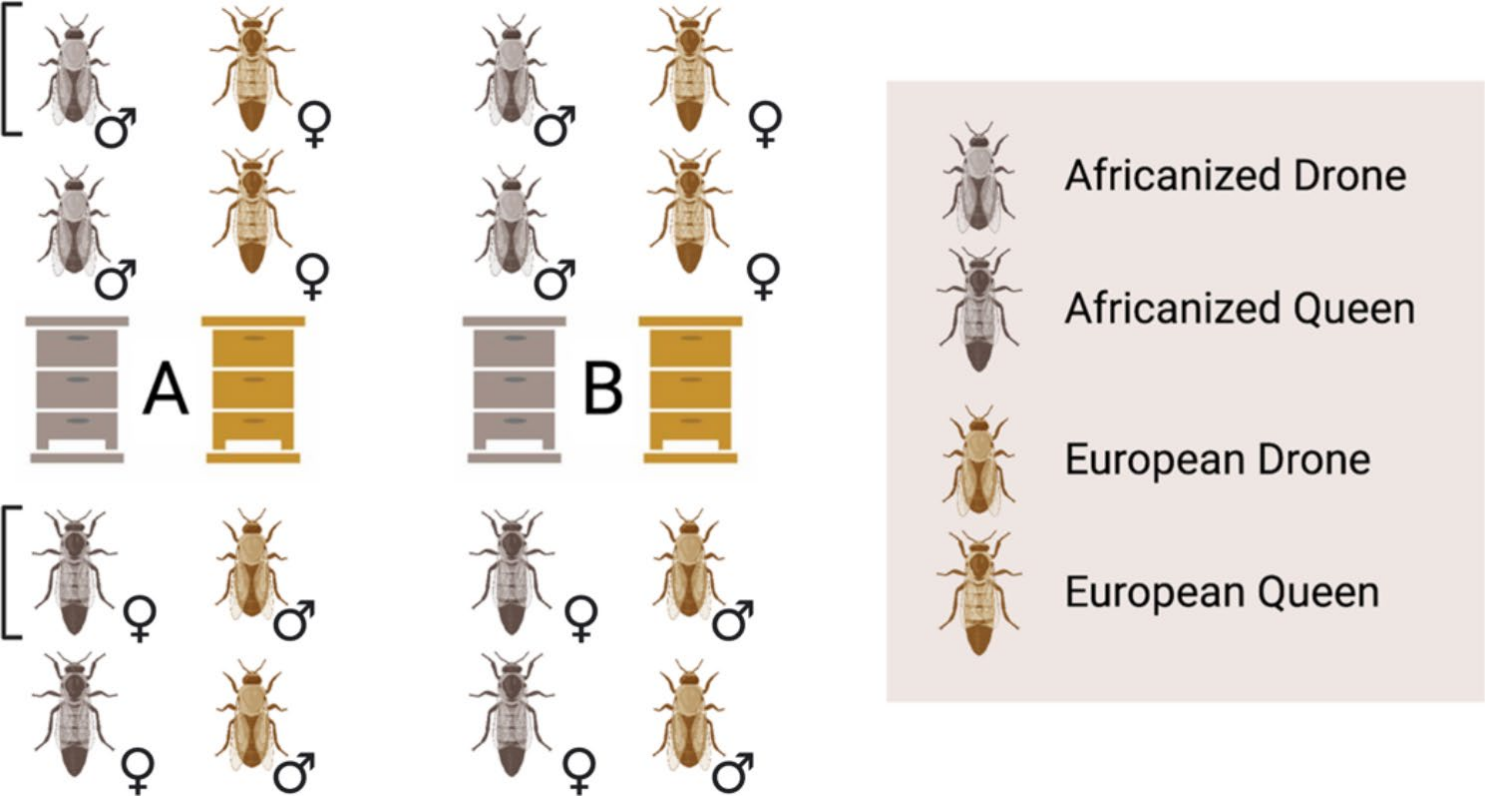
Breeding scheme used to generate samples for experiments. In Block A, queens and drones were derived from one Africanized (AHB) honey bee colony and one European (EHB) colony. One queen and drone from each colony was mated to create a reciprocal cross pair (an EHB queen was mated with an AHB drone, and an AHB queens was mated with an EHB drone). The worker offspring of the two crosses in a pair were placed in a common colony and used for behavioral assays and subsequently collected for molecular analysis. In Block A, workers from reciprocal cross 1 was placed in Colony 1, and reciprocal cross 2 was placed in Colony 2. A similar mating scheme was used in Block B to generate Colony 3 and Colony 4. Molecular analysis was performed on the workers generated from reciprocal cross 2 (marked with a bracket)

Reciprocally crossed colonies were generated in July 2019. We used two AHB colonies and two EHB colonies of *A. mellifera ligustica* stock managed at the Janice and John G. Thomas Honey Bee Facility of Texas A&M University (TAMU) in Bryan, TX. The genetic background of each colony was confirmed using a mitochondrial haplotype analysis [64]. The source colonies were separated into two blocks (“A” and “B”), with one AHB and one EHB colony assigned to each block. From each colony, queens were generated using a standard commercial practice known as “grafting” [86]. Labeled adult virgin (unmated) queens were housed in cages within a nursery colony until drones reared from those same colonies reached sexual maturity. Two queens and two drones from AHB colonies were selected from each colony and crossed with two queens and two drones from EHB colonies by instrumental insemination [87] to create two reciprocal cross pairs per block (Fig. 3). Inseminated queens were placed in their own colonies to initiate egg-laying, generating a total of four colonies with an AHB queen inseminated by a EHB drone, and four colonies with an EHB queen inseminated by an AHB drone. The entrances of these colonies were restricted with queen excluder material to prevent escape or further mating.

Approximately three weeks after the queens began laying fertilized eggs, we collected frames of sealed, emerging brood from each colony and stored them in separate containers within a standing incubator kept at constant temperature of 35^o^C and 75% relative humidity. The following day, approximately five hundred age-matched newly emerged workers from each cross were collected, marked on the thorax with non-toxic paint to identify their maternal lineage of origin, and combined with an equal number of bees from the reciprocal cross colony into “nucleus” hive boxes containing food but no queen or brood. In total, we created four small colonies, each containing 500 workers from each cross in a reciprocal cross design. We also added 1,000 unmarked bees from an unrelated colony to each hive to increase the number of workers in the nucleus colony. Workers were kept in a “queenright” state for the first eight days by providing a TempQueen strip (Mann Lake, Wilkes-Barre, PA) to the colony, as done previously [88]. Once the collection of workers was completed, the queen from each colony was collected and stored at − 80^o^C for DNA extraction and sequencing. Likewise, the bodies of the drones used for inseminating each queen were placed on dry ice and stored at − 80^o^C immediately after semen collection for subsequent analysis.

### Parent-of-origin effects on worker aggression

We performed an aggression assay following methods described in [89] on day 8 post-emergence, a point in adult worker bee life wherein individuals that are highly receptive to the alarm pheromone cue (soldier bees) have been demonstrated to undergo rapid induction of aggression [70]. The outer cover of each colony was repeatedly hit with a brick for 30 s to disturb the bees, and we continuously waved a swatch of black leather (approximately 10 cm x 10 cm) dosed with honey bee alarm pheromone (100 µL isopentyl-acetate; Sigma-Aldrich, St. Louis, MO) diluted at a 1:10 ratio with mineral oil (Sigma-Aldrich) in front of the colony entrance for 10 min. Colonies were set up approximately 20 m apart to prevent the spread of alarm pheromone to adjacent colonies. Worker bees that responded to the stimuli by stinging the leather swatch were collected as “aggressive” bees. Following the treatment, an equivalent number of labeled bees were collected from inside the colony as “non-aggressive” bees; these bees were likely nurse bees performing brood care duties but may have also included bees involved in food storing or comb-building. Forager bees were likely not inside the colony during the sampling period. Bees were collected immediately on dry ice, shipped to Central State University (CSU), and stored at − 80^o^C. The proportion of bees from each cross that responded to the stimuli were calculated, and logistic regression was conducted to test for an effect of parent lineage, treating colony and block as cofactors.

### Sample preparation for whole genome sequencing and Oxford nanopore direct RNA-seq

The queen and drone parents from each cross of one reciprocal cross pair in Block A (signified by the brackets in Fig. 3) were selected for whole genome sequencing (WGS). The thorax of each bee was removed, and the flight muscle tissue was dissected on a platform surrounded by dry ice to keep the tissue frozen until the DNA isolation procedure. Genomic DNA was isolated using a DNeasy Blood & Tissue Kit (Qiagen, Germantown, MD) and treated with 1 µL RNase A (NEB, Ipswich, MA). Samples were submitted to the Penn State Genomics Core for quantitation with a Qubit Fluorometer (Life Technologies, Carlsbad, CA) and quality control assessment by Genomic DNA ScreenTape analysis on a TapeStation 4150 (Agilent, Santa Clara, CA). WGS libraries were prepared using an Illumina DNA prep kit with unique 5’ and 3’ indexes, pooled, and sequenced on a NextSeq 2000 P3 flow cell to generate an average of 45 million 150 bp paired-end reads per sample (Supplementary Dataset 1, Table S1). WGS reads for this study are deposited in the NCBI Sequence Read Archive under SRA project accession PRJNA732718.

Three aggressive bees and three non-aggressive bees were randomly selected from each of the same crosses as above for sequencing. Heads and abdomens were dissected from each bee, and total RNA was isolated using an RNA Miniprep extraction kit (Zymo Research, Irvine, CA). Poly(A) RNA was isolated from approximately 15 µg total RNA using the NEBNext Poly(A) mRNA Magnetic Isolation Module (E7490, NEB, Ipswich, MA, USA) according to the manufacturer’s protocol with one adjustment: 60 µL magnetic oligo d(T)25 beads were used for the initial binding to accommodate the increased total RNA input. Immediately following isolation, library preparation from poly(A) RNA was performed using the Direct RNA Sequencing Kit (SQK-RNA002, Oxford Nanopore Technologies, Oxford, UK) and the manufacturer’s protocol (version DRS_9080_v2_revM_14Aug2019). One Nanopore flow cell (R9, Oxford Nanopore Technologies, Oxford, UK) was used for each sample. Flow cell check was performed to confirm 800 active pores immediately prior to using each flow cell, following the manufacturer’s protocol (Flow Cell Check protocol version PQE_1004_v1_revAC_06Jan2016). Flow cell priming was performed according to the manufacturers protocol for direct RNA sequencing. Data were acquired in the minKNOW UI version 4.1.22 using the default settings. Flow cells were run to depletion of the RNA input for approximately 25 h. Nanopore direct-seq reads for this study have been deposited the NCBI Sequence Read Archive in FAST5 format under SRA project accession PRJNA940795.

### Generation of parental transcriptomes

WGS reads were adapter trimmed with fastp [90], then aligned to the RefSeq *Apis mellifera* HAv3.1 genome assembly [91] with BWA-MEM [92] using the default settings. Variants were detected using freebayes [93] to account for differences in ploidy between queens and drones. Heterozygous variants, those with 0/0 genotype (indicating a quality below 1), and variants with QUAL < 30, were filtered using a custom R script utilizing the VariantAnnotation (v1.18.5) package [94], which is provided in the GitHub repository associated with this manuscript [95]. FAST5 files from all direct RNA-seq samples were processed with Guppy (v5.0.1.6) using the rna_r9.4.1_70bps_hac.cfg configuration (Oxford Nanopore Technologies) for base calling on a GPU. FASTQ files labeled “pass” were concatenated together to generate one FASTQ file per sample. Minimap (v2.21) [96] was used for alignment to the HAv3.1 genome assembly [91] in splice-aware mode with a kmer length of 14 using the RefSeq gene annotation (NCBI *Apis mellifera* Annotation Release 104) to guide alignment. SAMtools (v1.12) [97] was used to sort and index the resulting BAM files. Flair (v1.5) [98] was then used to convert BAM files to BED format, correct intron-exon junctions based on the gene annotation, and call isoforms across all samples. These isoforms included novel transcripts and genes in addition to published transcripts and genes. The filtered variants, along with the transcriptome output by Flair, were then used to generate parent-specific transcriptomes using a custom R script [95]. For each cross, the two parental transcriptomes were combined, with identical transcripts being labeled “both” and transcripts differing between parents being labeled with the parent ID. As expected, the majority of mitochondrial transcripts mapped to the maternal alleles in each cross (Supplementary Dataset 2, Figures S12-S13).

### Detection of allelic transcription and m6A differences

FAST5 files for all direct-seq samples were processed by Guppy (v3.1.5) (Oxford Nanopore Technologies) and the resulting FASTQ files labeled “pass” were concatenated together to generate one FASTQ per sample. Minimap 2 was used for alignment to the cross transcriptomes generated above. SAMtools was used to sort and index the resulting BAM files. We then used EpiNano [99] to calculate the read coverage and m6A probability at each site, combined with custom R scripts [95] to subset the EpiNano output to the A sites at the center of RRACH motifs. Read coverage and m6A probability at each position within published and novel transcripts, separated by allele, were then combined to generate read count and m6A probability matrices in R. Transcripts with *n* < 2 positions were filtered from the datasets prior to analyses of allele-biased transcription and m6A.

We utilized the Storer Kim binomial exact test of two proportions [61, 100] and a generalized linear mixed effects model with interaction terms (GLIMMIX) to identify genes that exhibited parent-of-origin allele-biased transcription following methods described in [17, 21, 22], and the two-tailed unpooled *z*-test to identify genes that exhibited parent-of-origin allele-biased m6A following methods described in [63]. A Storer-Kim test was conducted for each transcript for each SNP position to test for significant differences in the proportion of maternal and paternal read counts among the samples. Test results were then corrected to control for the FDR [101] and aggregated by transcript. For each transcript, all positions were required to exhibit the same direction of parent- or lineage-specific bias at previously established thresholds for *p1* (the proportion of reads mapping to the AHB allele in offspring from the EHB queen and AHB drone cross) and *p2* (the proportion of reads mapping to the AHB allele in offspring from the AHB queen and EHB drone cross) for the transcript to be reported as exhibiting bias. This design allows for differentiating between parent-of-origin bias (for example, a transcript exhibiting paternal bias has > 60% of reads aligning to the paternal allele in both crosses) and lineage-of-origin bias (for example, a transcript exhibiting AHB bias has > 60% of reads aligning to the paternal allele in individuals from the EHB mother x AHB father cross and > 60% of reads aligning to the maternal allele in individuals from the AHB mother x EHB father cross – i.e., > 60% of reads align to the nucleotide variant present in the AHB-lineage parent in both crosses) [61]. Additionally, a GLIMMIX [17, 21, 22] was fit for each transcript, separately, to test for an effect of parent, lineage, and their interaction. Test results were FDR corrected, and only transcripts with a significant effect of parent or lineage, but not their interaction, were considered to exhibit bias. Only genes considered to exhibit bias in both tests were reported as exhibiting bias. A two-tailed unpooled *z*-test of two proportions was conducted for each transcript for each SNP position to test for differences between maternal and paternal m6A probability among the samples [63]. *Z*-test results were then FDR corrected and aggregated by transcript. For each transcript, all positions were required to exhibit the same direction of parent- or lineage-specific bias as described above. Our reciprocal cross design and statistical framework allow for differentiating between random monoallelic expression (no consistent bias toward the paternal or maternal allele across samples [102, 103]), lineage-dependent expression (consistent bias toward either the EHB or AHB allele across samples) and parent-of-origin expression (consistent bias toward either the maternal or paternal allele across samples) [61].

### Detection of lincRNAs

Transcripts generated by Flair were defined as “intergenic” if they had no overlap with any annotated protein-coding, rRNA, miRNA, snRNA, snoRNA, tRNA, or guide RNA gene. Transcripts were retained for further analysis if they were non-mitochondrial, intergenic, non-viral, and were at least 200 nucleotides in length [104]. The findORFs function in the ORFhunterR [105] R package was used to identify putative open reading frames (ORFs) and their lengths. CPC2 [106] was used to identify transcripts with coding potential. Additionally, BLASTX [107] was used to align transcripts to the Uniprot and Swiss-Prot combined database [108] under default parameters and a required cutoff E-value of 0.001. The transcripts were searched against annotated rRNAs, snRNAs, snoRNAs, miRNAs, tRNAs, and gRNAs, in the NCBI RefSeq Amel HAv3.1 assembly using discontiguous megablast (from BLAST + v2.10.1). Transcripts were considered long intergenic non-coding RNAs (lincRNAs) if they met all the above criteria, did not appear to be protein-coding by any of the three above methods, and did not align to other types of non-coding RNA using discontiguous megablast.

### Detection of isoform switching

Flair was used to quantify the abundance of each transcript in each sample. Counts were then imported into IsoformSwitchAnalyzeR (v1.18.0) [109] in R to test for differential isoform transcription between aggressive and non-aggressive bees and between the crosses with DEXseq [110]. Transcripts detected to exhibit isoform switching at FDR < 0.1 were retained for further analysis. ORFs were then predicted *de novo* from the transcript sequences using IsoformSwitchAnalyzeR. Additionally, the transcript sequences were analyzed by CPC2 to predict coding potential, overriding ORFs called by IsoformSwitchAnalyzeR if the transcript was determined to be non-coding at a cutoff of 0.7. Hmmscan [111] was then used to compare predicted ORFs to the Pfam database [112] to predict protein domains. SignalP (v5.0) [113] was used to predict signal peptides within predicted ORFs. NetSurfP2 [114] was used to predict intrinsically disordered regions within predicted ORFs. Finally, alternative splicing was then analyzed within IsoformSwitchAnalyzeR, and isoform switches were determined to have “consequences” if there were changes in coding potential, domains, signal peptides, intrinsically disordered regions, or intron retention.

### Differential expression analyses

Flair was used for each cross to quantify transcript abundances for each sample for each gene. Counts were then summed within genes regardless of parent ID or transcript ID, and samples from both crosses were combined into one gene count matrix in R. Counts were then TMM normalized using the edgeR package [115], and genes with *n* < 8 counts per million in at least *n* = 3 samples were filtered. After filtering, TMM normalization was performed again on the raw counts to calculate normalized log_2_-based count per million values (logCPM) using the edgeR package with a prior count of 2 to stabilize fold-changes of lowly transcribed genes. Multidimensional scaling using the limma package [116] was used to assess the largest effects on gene composition among the samples, using the top 5,000 most variable genes between each pair of samples. Differential expression analysis was then performed using the limma-trend method to test for differences in transcript abundance between aggressive and non-aggressive bees and between the crosses. Test results were FDR corrected, and only genes with FDR < 0.05 were considered to exhibit differential expression.

## Electronic supplementary material

Below is the link to the electronic supplementary material.

Supplementary Material 1

Supplementary Material 2

## Data Availability

All analysis scripts for this study and detailed markdowns describing their use are available at https://github.com/sbresnahan/allele-specific-transcription-and-m6A [95]. WGS reads for this study are deposited in the NCBI Sequence Read Archive under SRA accession PRJNA732718, and Nanopore direct-seq reads are available in FAST5 format under SRA accession PRJNA940795.
