# Correction to: Examining parent-of-origin effects on transcription and RNA methylation in mediating aggressive behavior in honey bees (*Apis mellifera*)

**DOI:** 10.1186/s12864-024-11071-x

**Published:** 2024-12-20

**Authors:** Sean T. Bresnahan, Ellen Lee, Lindsay Clark, Rong Ma, Michael Markey, Juliana Rangel, Christina M. Grozinger, Hongmei Li-Byarlay

**Affiliations:** 1 Huck Institutes of the Life Sciences, Pennsylvania State University, University Park, USA; 2 Agricultural Research and Development Program, Central State University, Wilberforce, USA; 3 Department of Biochemistry and Molecular Biology, Wright State University, Dayton, USA; 4 HPCBio, University of Illinois at Urbana-Champaign, Champaign, USA; 5 Research Scientific Computing Group, Seattle Children’s Research Institute, Seattle, USA; 6 Department of Entomology, Texas A&M University, College Station, USA; 7 Department of Agricultural and Life Science, Central State University, Wilberforce, USA

**Correction to:** *BMC Genomics* **24, 315 (2023)**

10.1186/s12864-023-09411-4

Following publication of the original article it was reported that Michael Markey was missing from the authorship list.

The correct authorship list can be found in this Correction.

The original article has been updated.

