# Supplementary material for: Examining parent-of-origin effects on transcription and RNA methylation in mediating aggressive behavior in honey bees (Apis mellifera)
Source: BMC Genomics. 2023 Jun 12;24:315. doi: 10.1186/s12864-023-09411-4 (PMC10258952; doi:10.1186/s12864-023-09411-4)
Supplement: Supplementary file 1 — Supplementary Material 1 [file 12864_2023_9411_MOESM1_ESM.pdf]

Supplementary Dataset 2: Examining the role of transcription and post-transcriptional modifications on mediating intragenomic conflict in honey bees

Lindsay Clark, HPCBio, Roy J. Carver Biotechnology Center, University of Illinois, Urbana-Champaign
Sean Bresnahan, Huck Institutes of the Life Sciences, Pennsylvania State University

2023-03-13

Contents

Study design 1

Sample processing 1

Identification and differential expression of lincRNAs 2
QC on read counts . . . 3
Normalization and filtering . . . 4
Sample clustering . . . 8
Differential gene expression . . . 9
Heatmaps for differential lincRNA expression . . . 10

Analysis of isoform switching 11

QC and differential gene expression 12
Quality control . . . 12
Mitochondrial reads . . . 15
Normalization and filtering . . . 17
Sample clustering . . . 22
Differential gene expression . . . 22
Heatmaps for differential gene expression . . . 23

Models of the genes exhibiting splicing and isoform switching 25

Study design

This study uses whole genome resequencing data produced by Illumina DNA sequencing and transcriptome data produced by Oxford Nanopore direct RNA sequencing to understand behavioral differences between non-aggressive and aggressive honey bees. Two crosses in a reciprocal cross design between Africanized honey bees (AHB) and European honey bees (EHB) were made. Cross A had AHB as the female parent and EHB as the male parent. Cross B had EHB as the female parent and AHB as the male parent. Three or more aggressive bees and three or more non-aggressive bees (labeled T and C, respectively) were collected from each cross. Twelve head and abdomen samples, spanning multiple batches over the course of a year, were ultimately analyzed, with three samples per experimental group. RNA samples were sequenced with Oxford Nanopore Technology direct RNA sequencing on a FLO-MIN106 flowcell.

We test two main hypotheses:

1. Aggression is associated with an enrichment for paternal allele-biased transcription.
2. Allele-biased transcription is associated with allele-biased RNA m6A.

Additionally, we assess whether either allele-biased transcription or allele-biased RNA m6A are associated with differential gene expression and/or differential isoform usage.

All scripts and code for the full analyses are available at
https://github.com/sbresnahan/allele-specific-transcription-and-m6A.

Sample processing

FAST5 files from all samples were processed with Guppy 5.0.16 using the `rna_r9.4.1_70bps_hac.cfg` configuration (Oxford Nanopore Technologies) for base calling on a GPU. FASTQ files labeled "pass" were

concatenated together to generate one FASTQ per sample. Minimap 2.21[1] was used for alignment to the *Apis mellifera* HAv3.1 reference genome (plus common viral genomes) in splice-aware mode with a kmer length of 14, using the published gene annotation to guide alignment. SAMtools 1.12[2] was used to sort and index the resulting BAM files. Flair 1.5[3] was then used to convert BAM files to BED format and correct intron-exon junctions based on the published annotation, then call isoforms across all samples. These isoforms included published transcripts and genes as well as novel transcripts and genes.

Finally, EpiNano[4] was used to quantify read coverage and RNA m6A probability at parent SNPs within transcripts. combined with a custom R scripts to subset the EpiNano output to the A sites at the center of RRACH motifs. Read coverage and m6A probability at each position within published and novel transcripts, separated by allele, were then combined to generate read count and m6A probability matrices.

Identification and differential expression of lincRNAs

Of the 31,475 transcripts output by Flair[5], 451 were already annotated as long non-coding RNAs (lncRNAs) in the NCBI GCF_003254395.2_Amel_HAv3.1 annotation, while 12 were for genes annotated as ribosomal RNA (rRNA) or small nuclear RNA (snRNA), and 17,880 were for protein-coding genes. Although snoRNAs, miRNAs, tRNAs, and guide RNAs were annotated in the reference genome, Flair did not output transcripts for any of these.

Transcripts were defined as "intergenic" if they had no overlap with any annotated protein-coding, rRNA, miRNA, snRNA, snoRNA, tRNA, or guide RNA gene; 3,908 transcripts were considered intergenic by this measure. Additionally, 77 transcripts were on the mitochondrial genome (including transcripts matching gene NC_037641.1:1186000). Transcripts were retained for further analysis if they were non-mitochondrial, intergenic, and were at least 200 nucleotides in length, leaving 3806 transcripts for further analysis, including 6 transcripts that were later removed for being viral in origin.

The `findORFs` function in the ORFhunterR[6] R package was used to identify putative open reading frames (ORFs) and their lengths. However, 142 of the 165 annotated lncRNAs that were analyzed had putative ORFs using this method, so this information was ignored in favor of other approaches for determining if transcripts were protein-coding. Using CPC2[7], 61 out of 3806 transcripts were determined to have coding potential. Additionally, BLASTX (from BLAST+ v2.10.1) was used to align transcripts to the Uniprot and Swiss-Prot combined database (from a June 2020 mirror) under default parameters. Using a cutoff E value of 0.001, 120 transcripts aligned to a known protein sequence. Lastly, gene sequences for all annotated snRNA, snoRNA, miRNA, tRNA, rRNA, and guide RNAs were extracted from the GCF_003254395.2_Amel_HAv3.1 reference sequence. Since there were no annotated 7SL RNA genes in the reference, we used sequences NR_001992.2 and NR_037753.2 from *Drosophila melanogaster* to represent those. We then aligned the putative lincRNA transcripts to all of these other non-coding RNAs using discontiguous megablast (from BLAST+ v2.10.1) under default parameters, finding six transcripts aligning very strongly to rRNA genes.

Altogether, 3642 transcripts were retained as lincRNAs for being non-mitochondrial, non-viral, intergenic, at least 200 nt long, not predicted to have coding potential by CPC2, not aligning to known proteins, and not

[1] Li, H. (2018). Minimap2: pairwise alignment for nucleotide sequences. Bioinformatics, 34:3094-3100. doi:10.1093/bioinformatics/bty191

[2] Petr Danecek, James K Bonfield, Jennifer Liddle, John Marshall, Valeriu Ohan, Martin O Pollard, Andrew Whitwham, Thomas Keane, Shane A McCarthy, Robert M Davies, Heng Li (2021). Twelve years of SAMtools and BCFtools. GigaScience, Volume 10, Issue 2, giab008, https://doi.org/10.1093/gigascience/giab008

[3] Tang, A.D., Soulette, C.M., van Baren, M.J. et al. Full-length transcript characterization of SF3B1 mutation in chronic lymphocytic leukemia reveals downregulation of retained introns. Nat Commun 11, 1438 (2020). https://doi.org/10.1038/s41467-020-15171-6

[4] Liu, H., Begik, O., Lucas, M.C. et al. Accurate detection of m6A RNA modifications in native RNA sequences. Nat Commun 10, 4079 (2019). https://doi.org/10.1038/s41467-019-11713-9

[5] Tang, A.D., Soulette, C.M., van Baren, M.J. et al. Full-length transcript characterization of SF3B1 mutation in chronic lymphocytic leukemia reveals downregulation of retained introns. Nat Commun 11, 1438 (2020). https://doi.org/10.1038/s41467-020-15171-6

[6] Grinev V, Yatskou M, Skakun V, Chepeleva M, Nazarov P (2021). ORFhunteR: Predict open reading frames in nucleotide sequences. R package version 1.2.0.

[7] Kang Y. J., Yang D. C., Kong L., Hou M., Meng Y. Q., Wei L., Gao G. 2017. CPC2: a fast and accurate coding potential calculator based on sequence intrinsic features. Nucleic Acids Research 45(Web Server issue): W12–W16.

aligning to other non-coding RNAs. Tallies of annotated and novel lincRNAs and their corresponding genes are listed in Table S1.

Table S 1: Tallies of annotated and unannotated lincRNAs after filtering.

Gene_type	Genes	Annotated_lncRNAs	Annotated_pseudogene_RNAs	Novel_RNAs
Annotated	219	158	1	116
Novel	2970	0	0	3367

QC on read counts

Out of the 3642 lincRNA transcripts identified, Flair output read counts for 3640 of them (Fig. S1). The proportion of reads mapping to lincRNAs varied among samples, with T samples having slightly lower proportions than C samples (P = 0.055). At a gross scale the total number of lincRNA reads followed the total library size (Fig. S2).

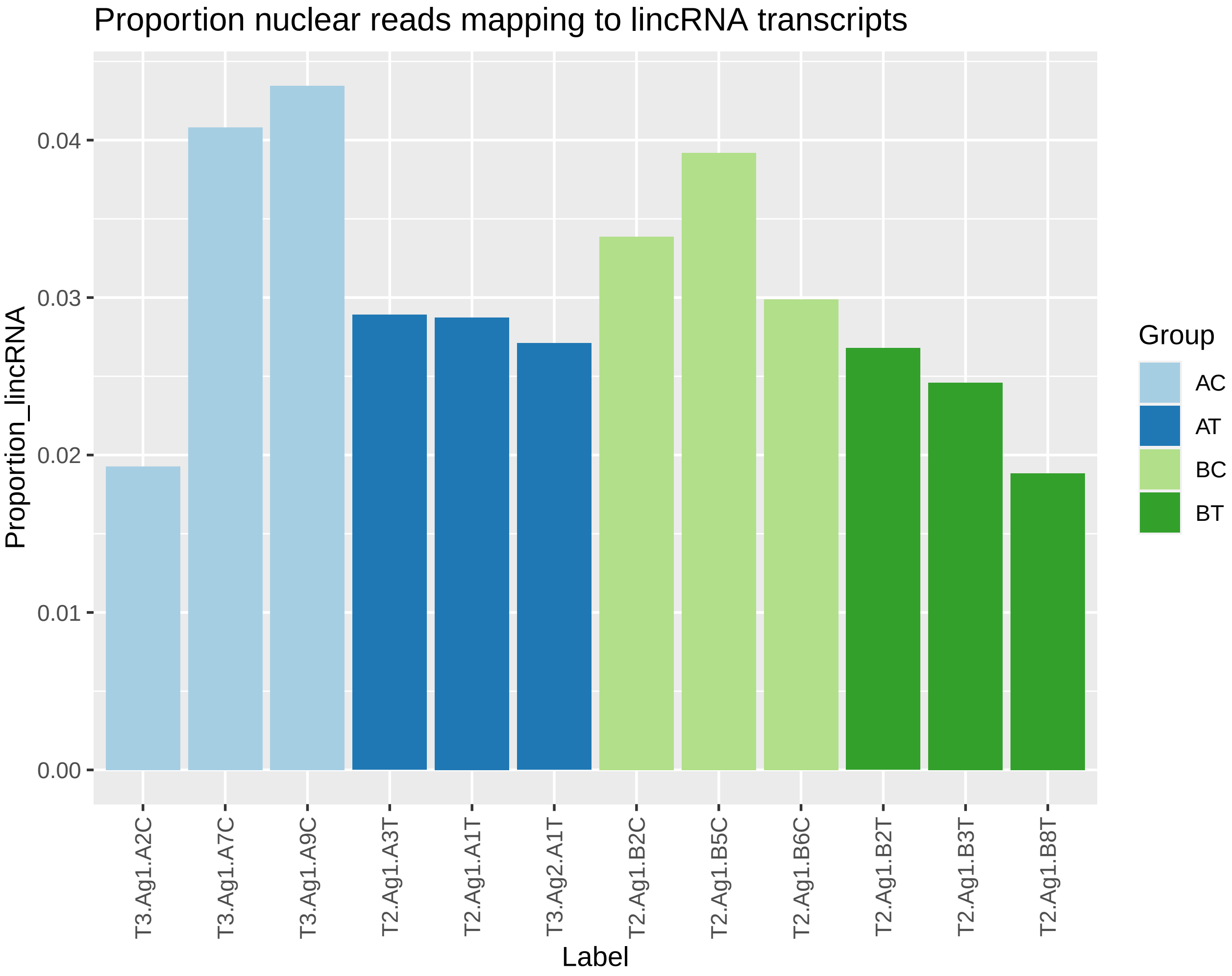

Figure S1: Proportion of reads mapping to lincRNAs, out of total reads mapping to the nuclear genome. Reads mapping to mitochondrial or viral genomes were excluded.

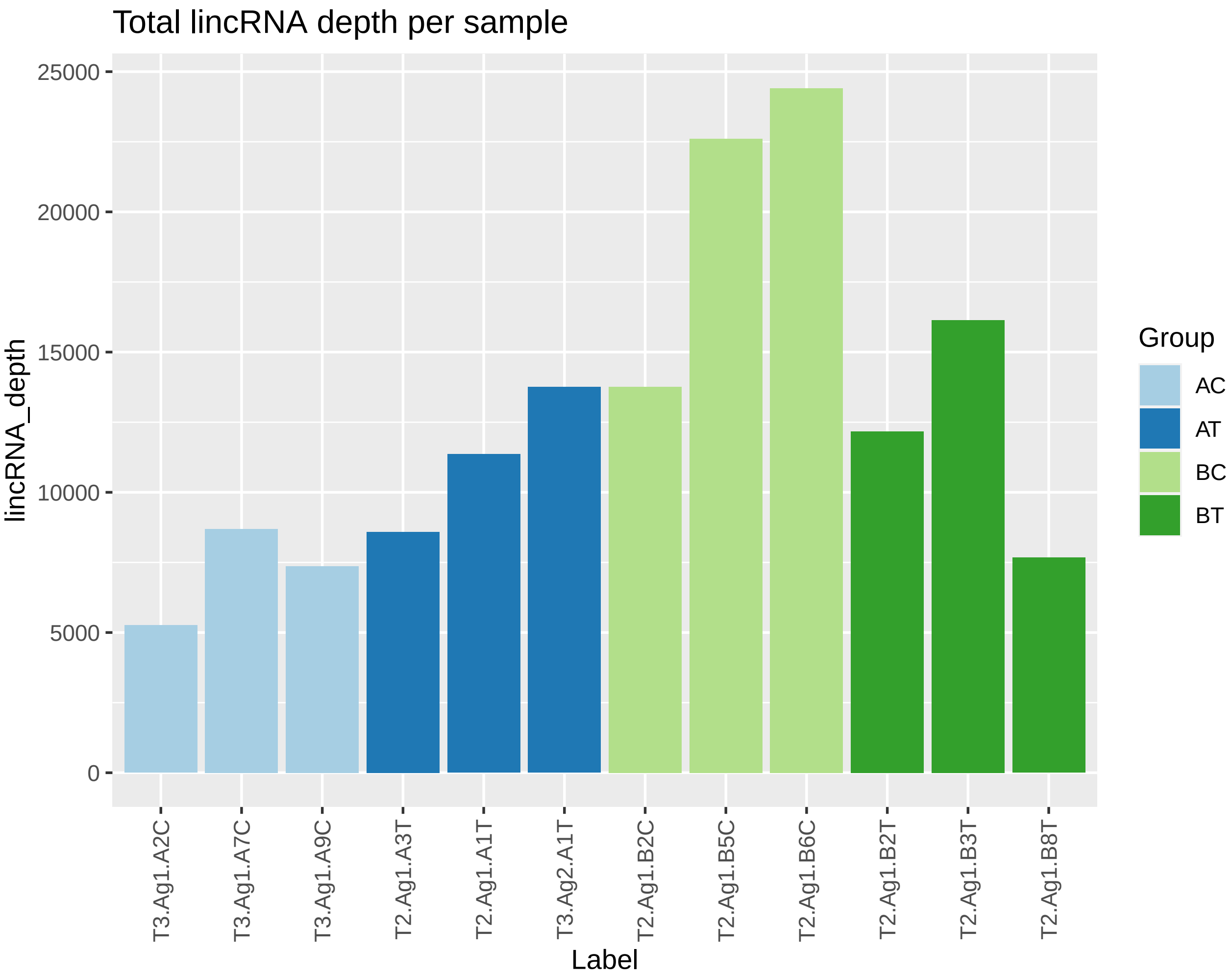

Figure S2: Total number of reads mapping to lincRNAs in each sample.

Normalization and filtering

When comparing expression levels, the numbers of reads per transcript need to be normalized not only because of the differences in total number of reads, but because there could be differences in RNA composition such that the total number of reads would not be expected to be the same. The TMM (trimmed mean of M values) normalization[8] in the edgeR package[9] uses the assumption of *most genes do not change* to calculate a normalization factor for each sample to adjust for such biases in RNA composition. In this dataset, TMM normalization factors fluctuated between 0.84 and 1.3. TMM normalization factors are multiplied by the library size in order to generate the effective library size, where smaller TMM normalization factors indicate more overrepresented genes in the sample. Normalization factors varied among samples but were not particularly associated with experimental group (Fig. S3).

[8]Robinson MD, Oshlack A (2010). A scaling normalization method for differential expression analysis of RNA-seq data. Genome Biology 11, R25.

[9]Robinson MD, McCarthy DJ, Smyth GK (2010). "edgeR: a Bioconductor package for differential expression analysis of digital gene expression data." Bioinformatics, 26(1), 139-140.

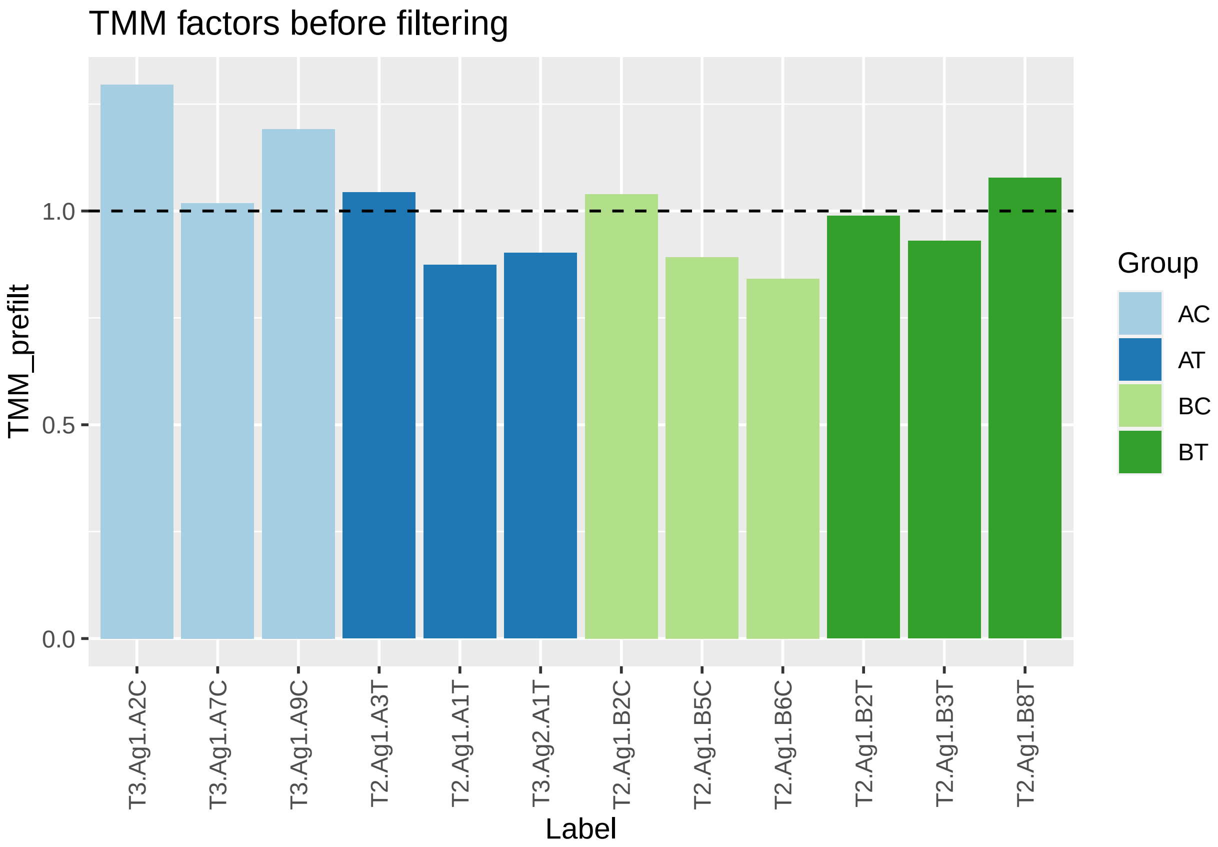

Figure S3: Normalization factors before filtering

While the sequence analysis identified a total of 3,640 lincRNA transcripts, many of these might not have detectable expression in enough samples for differential expression to be detectable. Therefore, we set the detection threshold at 250 cpm (counts per million) in at least 3 samples, which resulted in 1,225 lincRNAs being filtered out, leaving 2,415 lincRNAs to be analyzed for differential expression, containing 96.54% of the reads. This threshold resulted in a fairly even number of genes being detected across samples, considering the variability in library size (Fig. S4). In a typical Illumina RNA-seq experiment, we might use 1 cpm as the filtering threshold. Given the lower read depth of this experiment due to the use of Oxford Nanopore sequencing and filtering down to lincRNAs, a higher threshold was needed to differentiate between lincRNAs that could be detected in few samples vs. lincRNAs that could be detected in most samples. We arrived at a threshold of 250 by examining the detection rates at a variety of thresholds ranging from 200 to 500, where 164 CPM corresponded to zero reads in the raw data (results not shown).

After filtering, TMM normalization was performed again and normalized log2-based count per million values (logCPM) were calculated using edgeR's[10] `cpm()` function with `prior.count = 2` to help stabilize fold-changes of extremely low expression genes. TMM normalization factors after filtering are shown in Fig. S5, and compared to pre-filtering values in Fig. S6.

[10] McCarthy DJ, Chen Y, Smyth GK (2012). "Differential expression analysis of multifactor RNA-Seq experiments with respect to biological variation." Nucleic Acids Research, 40(10), 4288-4297.

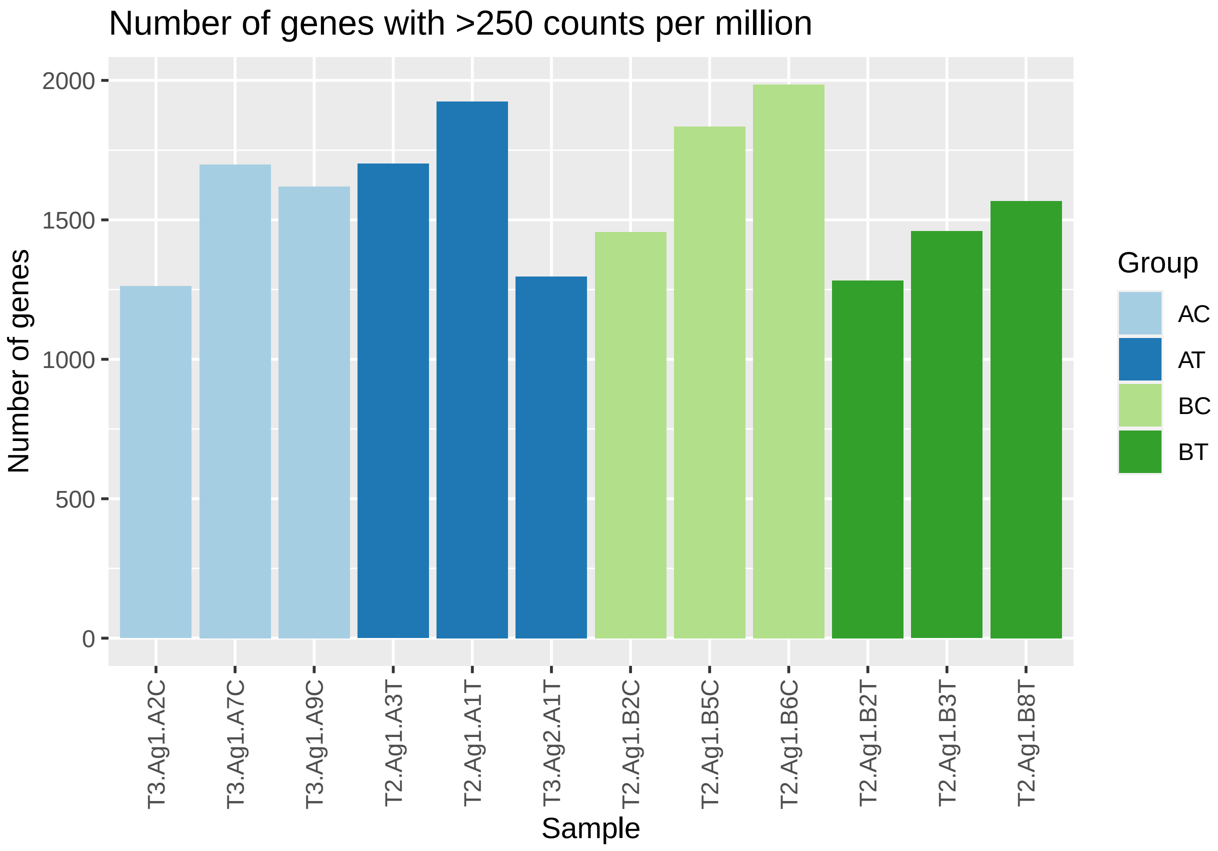

Figure S4: Number of detected genes passing the filtering threshold in each sample.

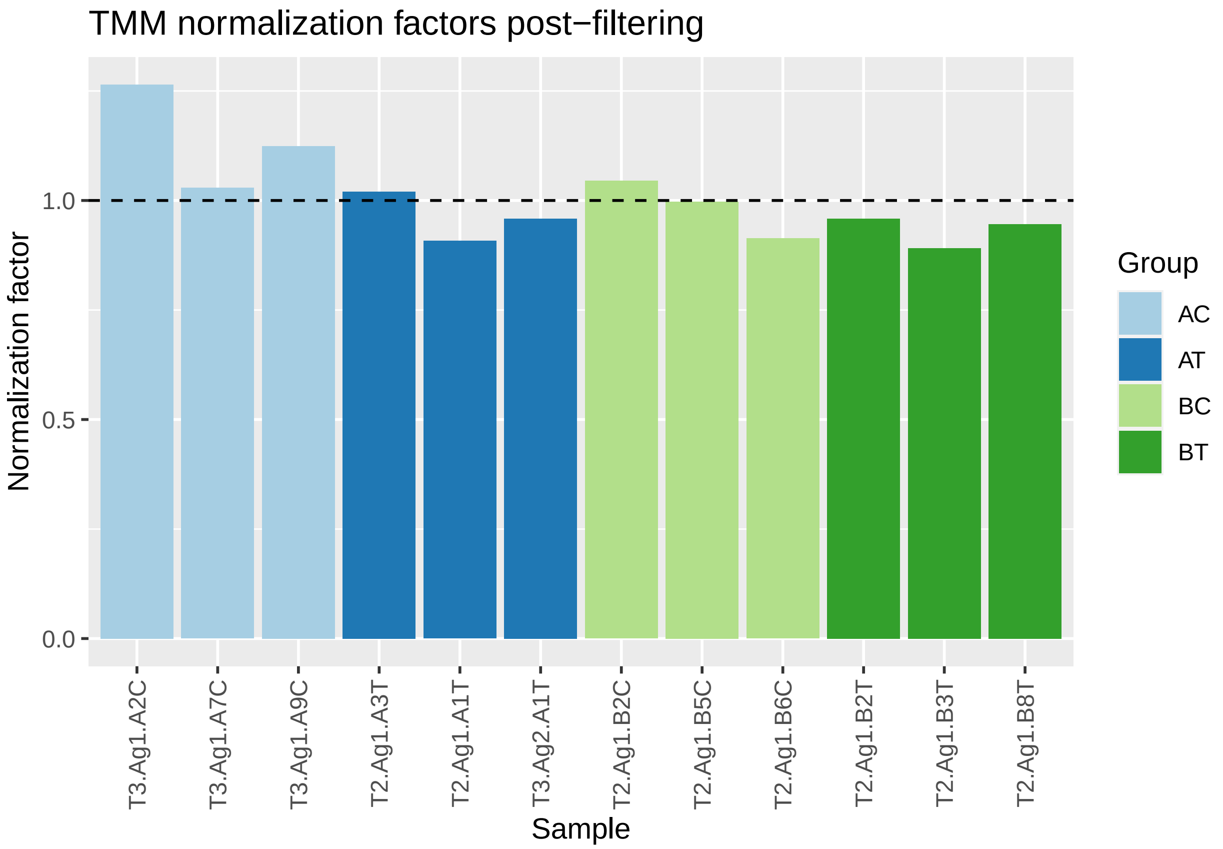

Figure S5: TMM normalization factors post-filtering.

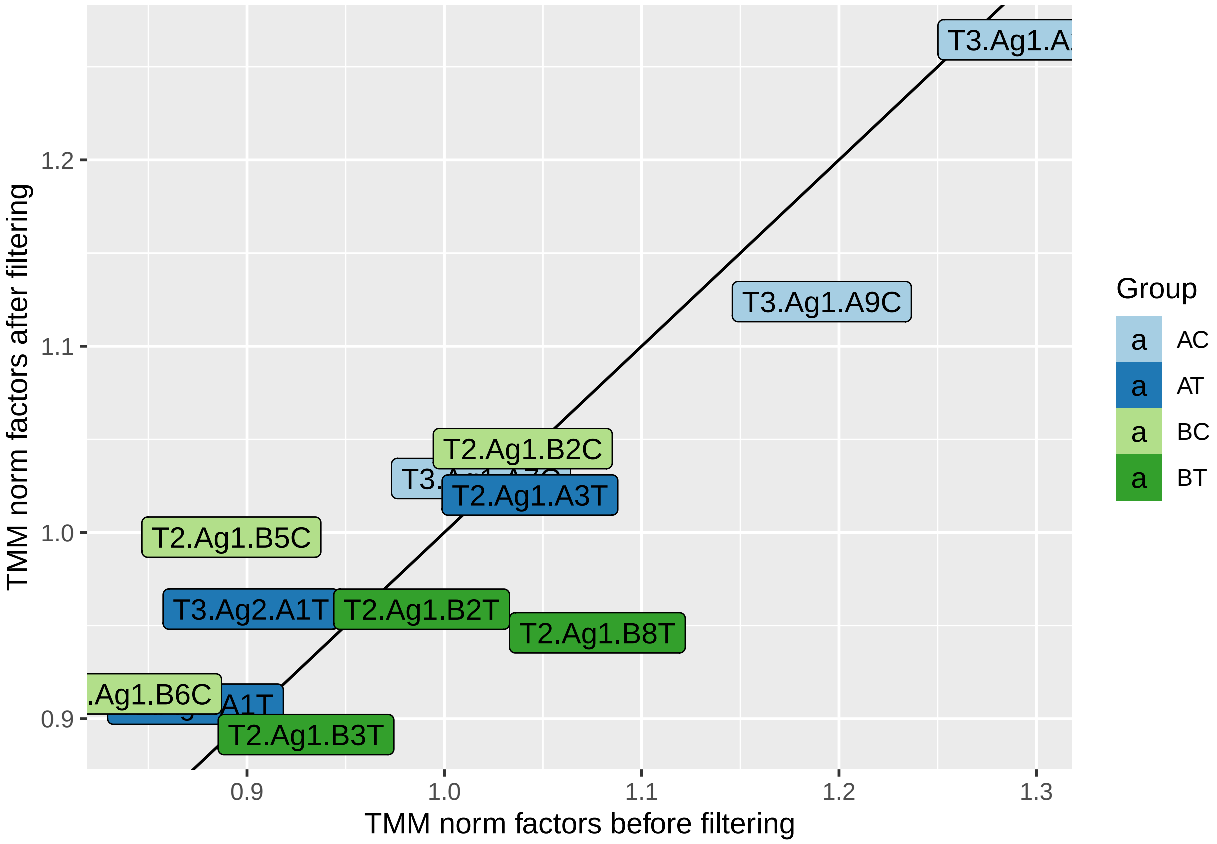

Figure S6: Comparison of TMM normalization factors before and after filtering.

Sample clustering

Multidimensional scaling in the limma[11] package was used to assess the largest effects on gene composition among samples. The normalized logCPM values from all 2415 lincRNAs between each pair of samples were chosen to construct the multidimensional scaling plot. The first axis separated the two crosses, while the second axis separated C and T samples (Fig. S7).

[11]Ritchie, M.E., Phipson, B., Wu, D., Hu, Y., Law, C.W., Shi, W., and Smyth, G.K. (2015). limma powers differential expression analyses for RNA-sequencing and microarray studies. Nucleic Acids Research 43(7), e47.

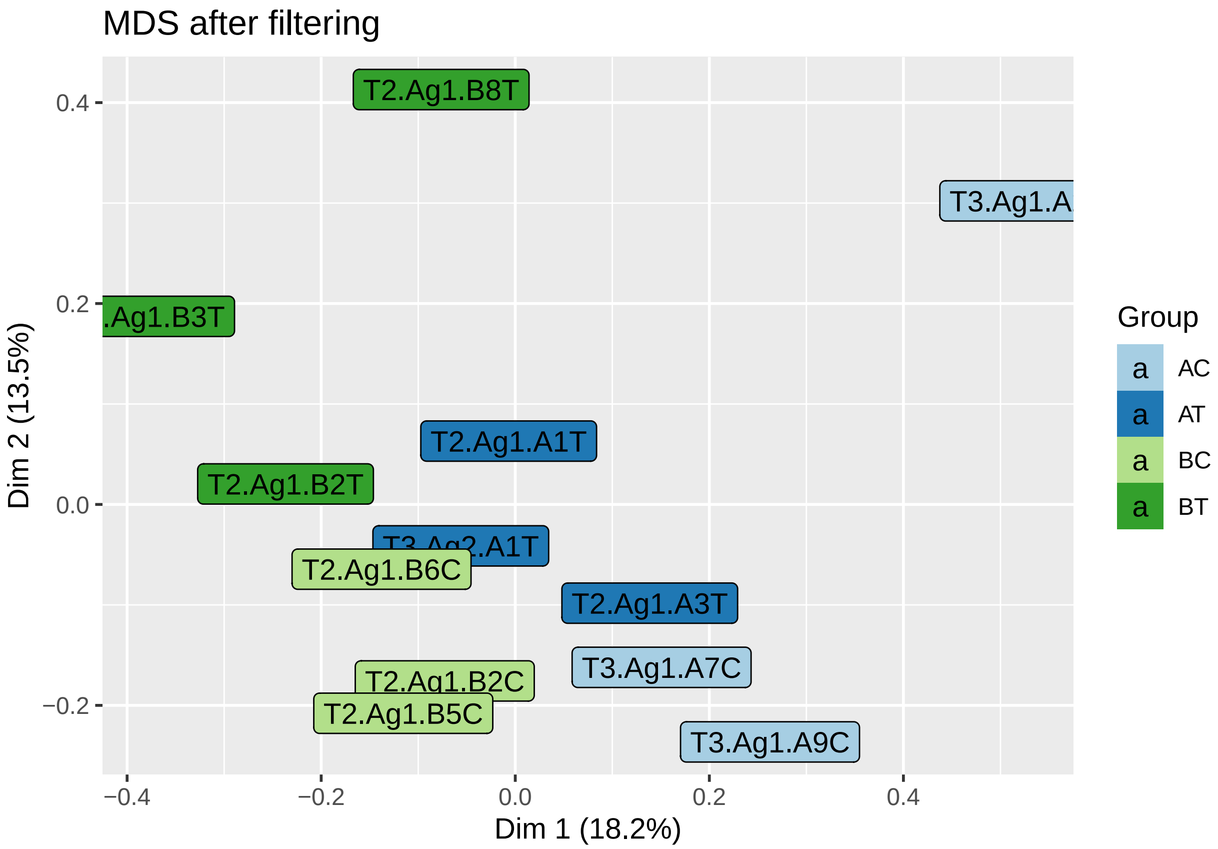

Figure S7: Multidimensional scaling of samples after filtering, using the top 5000 variable genes between each pair of samples.

Differential gene expression

Differential gene expression (DE) analysis was performed using the limma-trend method[12]. Multiple testing correction was done using the False Discovery Rate method[13]. The model run was:

$Y = Group$

where *Group* refers to the AC, AT, BC, and BT groups. The following contrasts were then assessed:

- **BT - BC**: Differences between T and C bees from the B cross
- **AT - AC**: Differences between T and C bees from the A cross
- **BC - AC**: Differences between B and A crosses, with C as the baseline
- **BT - AT**: Differences between B and A crosses in T samples
- **(BT + AT) - (BC + AC)**: Differences between T and C bees across both crosses
- **(BT + BC) - (AT + AC)**: Differences between crosses, looking at both C and T
- **(BT - BC) - (AT - AC)**: Interaction effect; differential effect of cross on differences between T and C bees

Numbers of significantly up- and down-regulated genes are shown in Table S2.

[12] Chen Y, Lun ATL and Smyth GK. From reads to genes to pathways: differential expression analysis of RNA-Seq experiments using Rsubread and the edgeR quasi-likelihood pipeline [version 2; referees: 5 approved]. F1000Research 2016, 5:1438 (doi: 10.12688/f1000research.8987.2)

[13] Benjamini, Y., & Hochberg, Y. (1995). Controlling the false discovery rate: a practical and powerful approach to multiple testing. Journal of the Royal statistical society: series B (Methodological), 57(1), 289-300.

Table S 2: Number of gene significant for differential expression at FDR < 0.05.

	BT - BC	AT - AC	BC - AC	BT - AT	(BT + AT) - (BC + AC)	(BT + BC) - (AT + AC)	(BT - BC) - (AT - AC)
**Down**	**1**	**0**	**1**	**4**	**3**	**2**	**0**
NotSig	2409	2413	2404	2408	2401	2396	2415
**Up**	**5**	**2**	**10**	**3**	**11**	**17**	**0**

Results for all genes are listed in Supplementary Dataset S1.

Heatmaps for differential lincRNA expression

Across all of the experimental groups contrasts, a one-way ANOVA was run in limma, identifying 41 significant lincRNAs. These lincRNA were then plotted in a heatmap to summarize gross changes in lincRNA expression across groups. (Fig. S8).

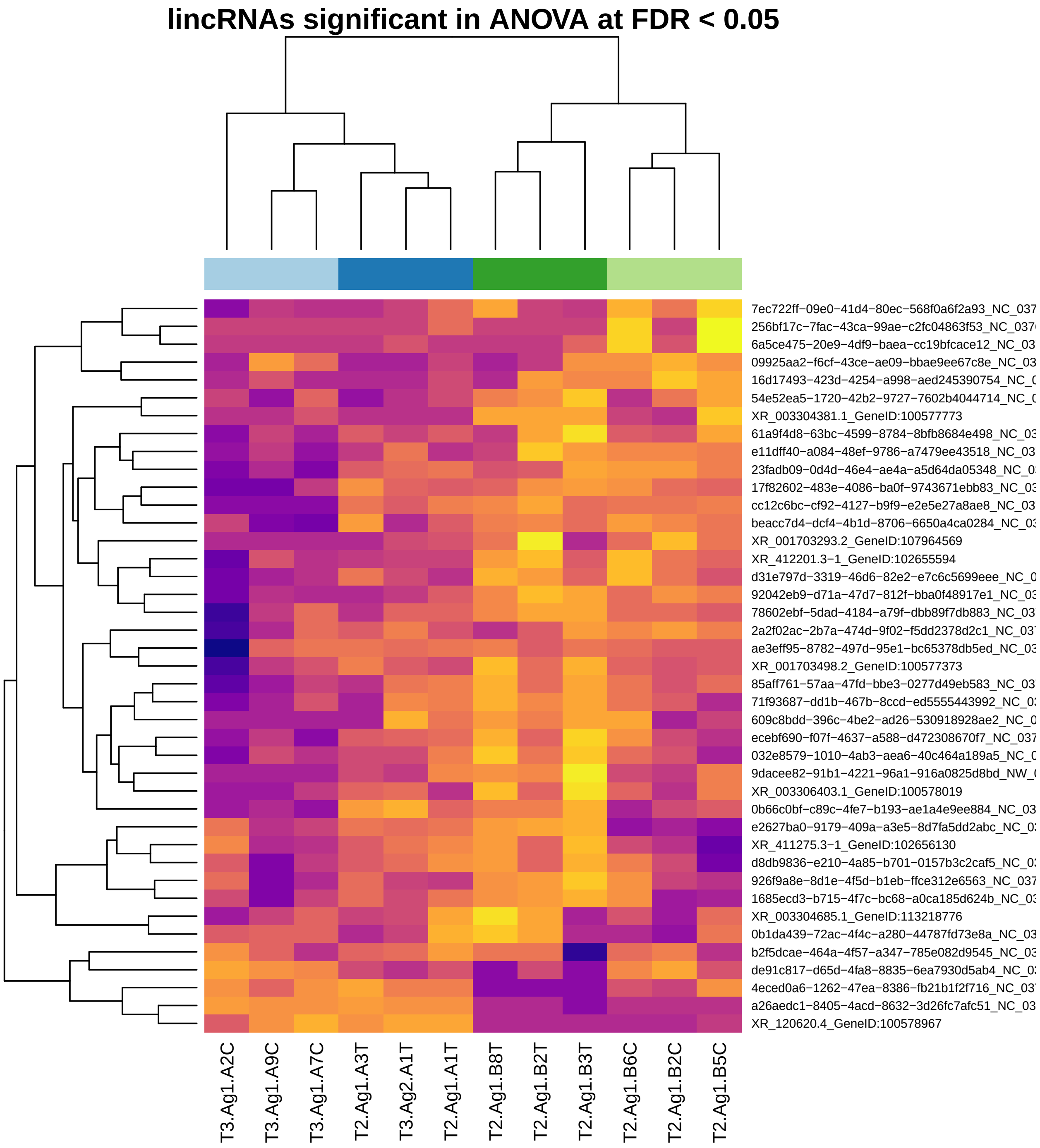

Figure S8: Heatmap of 29 lincRNAs with FDR < 0.05 in one-way anova for effect of experimental group.

Analysis of isoform switching

Few genes were significant for isoform switching, and those that were tended to be highly expressed, suggesting that more significant switches could have been detected with higher sequence read depth. However, the switches that were detected are potentially interesting, for example the switch from a coding transcript to a non-coding transcript for *Apid1* in cross B as compared to cross A, as well as group BC as compared to BT

(see PDFs of switch consequences). Genes with significant switches with putative functional consequences are listed in Table S3. With respect to the analysis before fixing the flair bug, more significant switches with functional consequences were found for most contrasts.

Table S 3: Genes with significant (FDR < 0.1) isoform switches with putative functional consequences.

	gene_id	gene_name	condition_1	condition_2	gene_switch_q_value
1	GeneID:406140	Apid1	AC	BC	0.0000000
3	GeneID:406140	Apid1	BC	BT	0.0000000
7	GeneID:406140	Apid1	AT	BT	0.0000000
8	GeneID:406121	Mrjp3	AT	BT	0.0000000
10	NC_001566.1:13000	NA	AC	BC	0.0000506
13	GeneID:406140	Apid1	A	B	0.0001549
2	GeneID:406121	Mrjp3	A	B	0.0002034
110	GeneID:409924	LOC409924	C	T	0.0004062
31	GeneID:409299	LOC409299	C	T	0.0008968
11	GeneID:406121	Mrjp3	AC	BC	0.0018311
4	NC_037644.1:5664000	NA	A	B	0.0025668
5	GeneID:552829	LOC552829	A	B	0.0029525
12	GeneID:725215	LOC725215	BC	BT	0.0032881
14	GeneID:409924	LOC409924	BC	BT	0.0080161
15	GeneID:727037	LOC727037	AT	BT	0.0116237
16	GeneID:410806	LOC410806	AT	BT	0.0199640
71	GeneID:724565	LOC724565	A	B	0.0335776
81	GeneID:413141	LOC413141	A	B	0.0553019
17	GeneID:413141	LOC413141	AT	BT	0.0709019
18	GeneID:727037	LOC727037	BC	BT	0.0813682
19	GeneID:551806	LOC551806	BC	BT	0.0852233
51	GeneID:413069	LOC413069	C	T	0.0905798
6	GeneID:551459	LOC551459	C	T	0.0905798
82	GeneID:551763	LOC551763	C	T	0.0905798

QC and differential gene expression

Quality control

Libraries varied considerably in overall size (Fig. S9) but were similar in quality (Fig. S10). Two samples had low levels of viral contamination (Fig. S10). Most reads mapping to the nuclear genome mapped to annotated transcripts (Fig. S10). Number of genes detected was linearly correlated with library size, indicating that saturation of gene detection was not reached (Fig. S11). The A-C group, which consisted entirely of new samples from December 2021, had the smallest library size and lowest number of genes detected (Figs. S9 and S11).

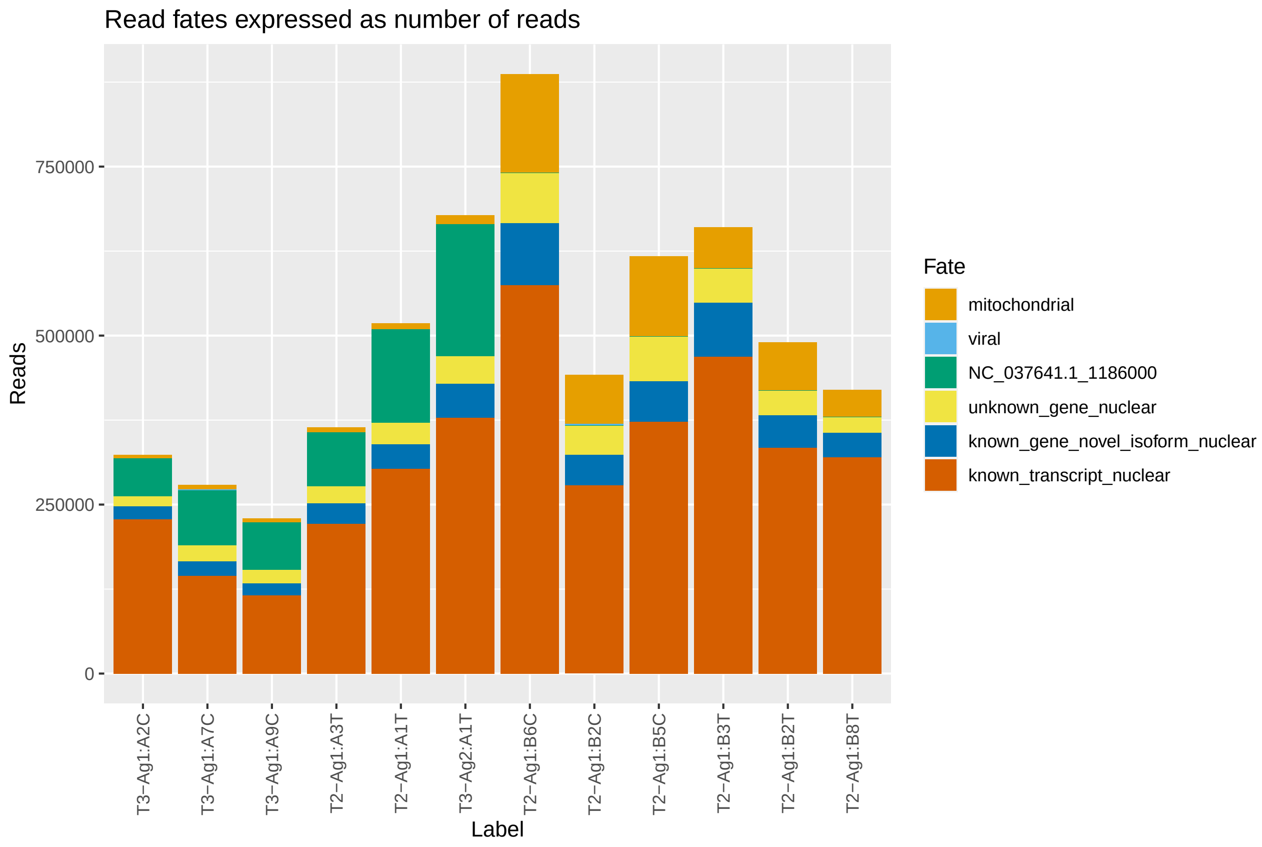

Figure S9: Library sizes expressed as number of aligned reads.

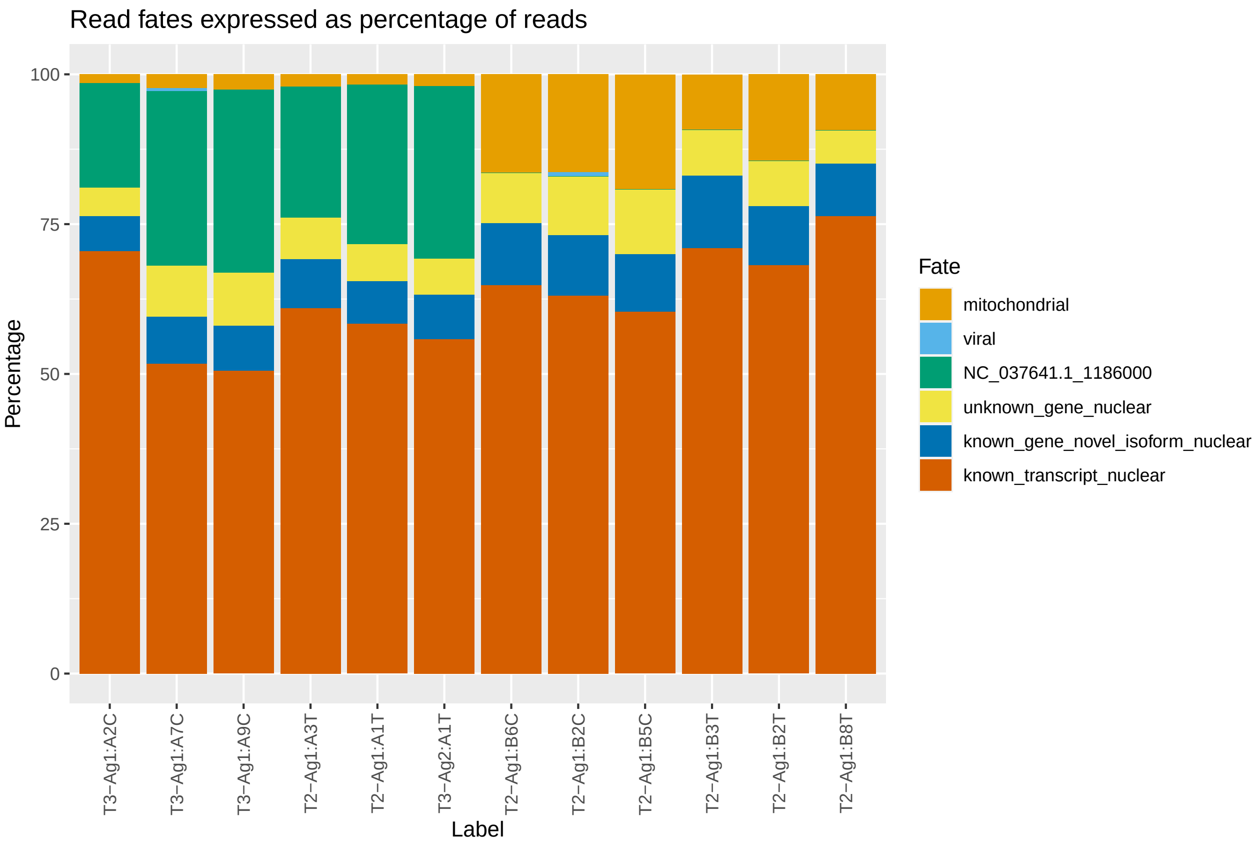

Figure S10: Percentages of reads aligned to categories of transcripts.

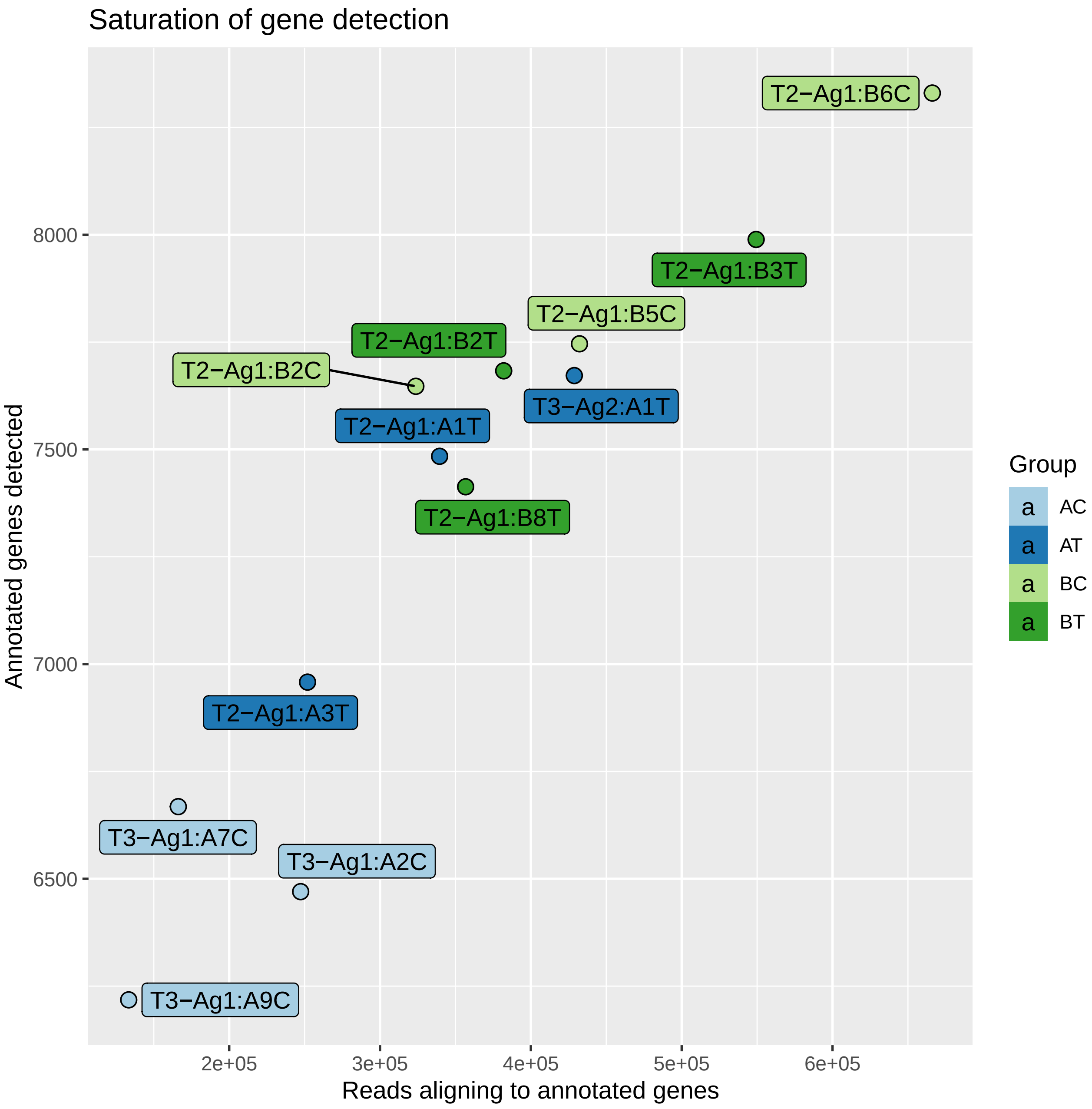

Figure S11: Number of genes detected vs. number of reads aligning to genes. Only genes from the published annotation are considered.

Mitochondrial reads

The vast majority of reads mapping to mitochondrial transcripts mapped correctly to the mother in both crosses (Figs. S12 and S13). Because of the mismapping of mitochondrial reads to the nuclear genome (NC_037641.1:1186000) in cross A, we excluded from downstream analysis all mitochondrial reads including those mapping to NC_037641.1:1186000. We found that this filtering reduced variance in TMM normalization factors (data not shown).

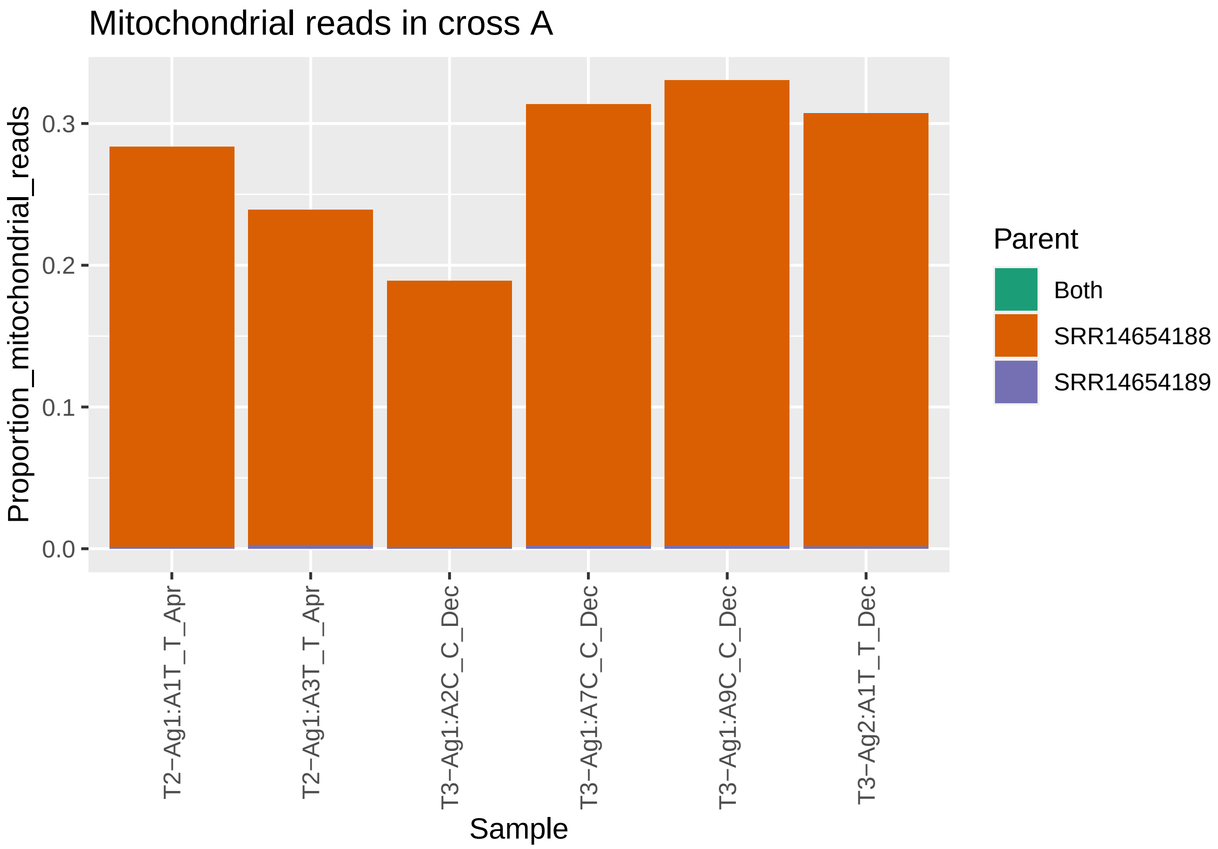

Figure S12: Proportion of read assigned to the materinal mitochondrion (SRR14654188), paternal mitochondrion (SRR14654189) or mitochondrion of unknown parent (Both) in cross A. Reads assigned to gene NC_037641.1:1186000 are counted as mitochondrial.

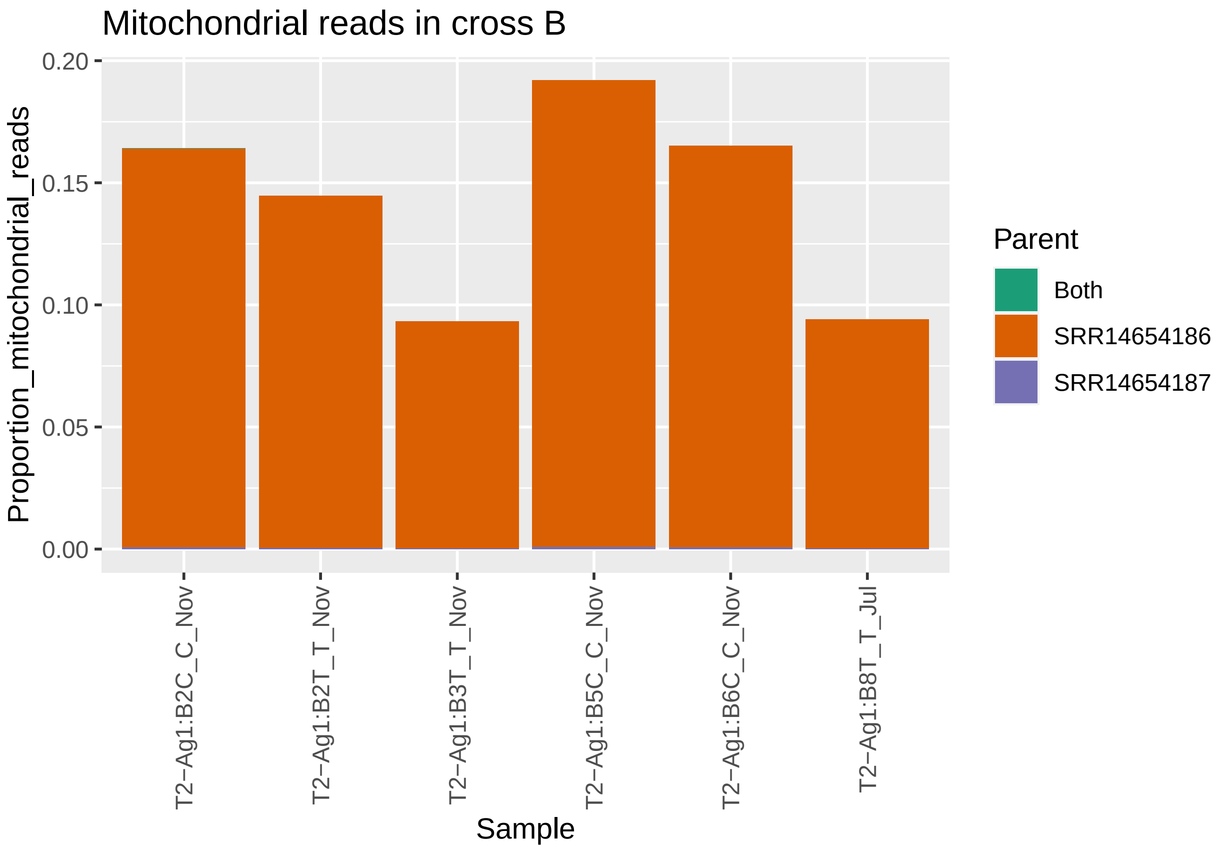

Figure S13: Proportion of read assigned to the materinal mitochondrion (SRR14654186), paternal mitochondrion (SRR14654187) or mitochondrion of unknown parent (Both) in cross B. Reads assigned to gene NC_037641.1:1186000 are counted as mitochondrial.

Normalization and filtering

When comparing expression levels, the numbers of reads per gene need to be normalized not only because of the differences in total number of reads, but because there could be differences in RNA composition such that the total number of reads would not be expected to be the same. The TMM (trimmed mean of M values) normalization[14] in the edgeR package[15] uses the assumption of *most genes do not change* to calculate a normalization factor for each sample to adjust for such biases in RNA composition. In this dataset, TMM normalization factors fluctuated between 0.79 and 1.21. TMM normalization factors are multiplied by the library size in order to generate the effective library size, where smaller TMM normalization factors indicate more overrepresented genes in the sample. Normalization factors varied among samples but were not particularly associated with experimental group (Fig. S14).

[14] Robinson MD, Oshlack A (2010). A scaling normalization method for differential expression analysis of RNA-seq data. Genome Biology 11, R25.

[15] Robinson MD, McCarthy DJ, Smyth GK (2010). “edgeR: a Bioconductor package for differential expression analysis of digital gene expression data.” Bioinformatics, 26(1), 139-140.

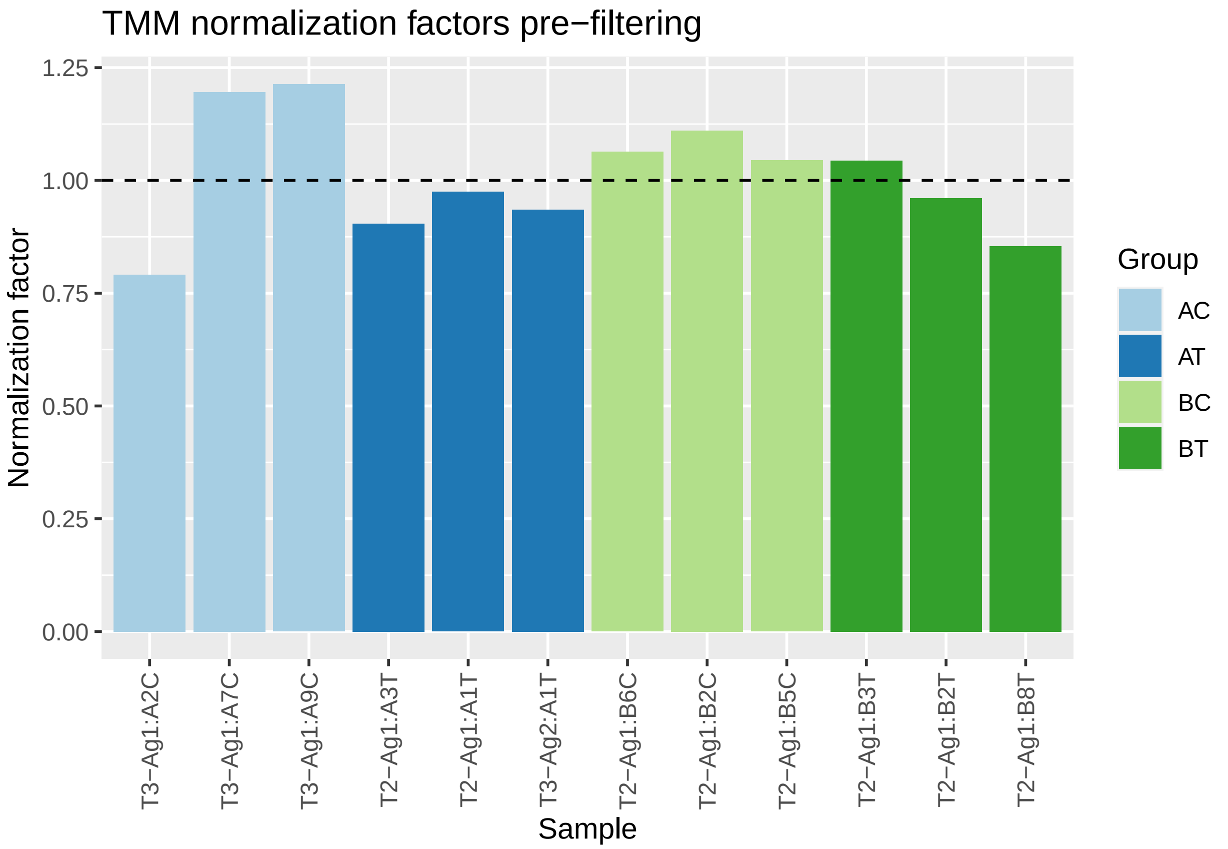

Figure S14: TMM normalization factors pre-filtering.

While the `flair collapse` output a total of 19,902 genes (after filtering mitochondrial genes), many of these might not have detectable expression in these samples. Therefore, we set the detection threshold at 8 cpm (counts per million) in at least 3 samples, which resulted in 5,440 genes being filtered out, leaving 14,462 genes to be analyzed for differential expression, containing 99.42% of the reads. Of these genes, 8058 were from the published annotation, representing 90.30% of reads prior to filtering. This threshold resulted in a fairly even number of genes being detected across samples, considering the variability in library size (Fig. S15). In a typical Illumina RNA-seq experiment, we might use 1 cpm as the filtering threshold. Given the lower read depth of this experiment due to the use of Oxford Nanopore sequencing, a higher threshold was needed to differentiate between genes that could be detected in few samples vs. genes that could be detected in most samples. We arrived at a threshold of 8 by examining the detection rates at a variety of thresholds ranging from 4 to 12 (results not shown).

After filtering, TMM normalization was performed again and normalized log2-based count per million values (logCPM) were calculated using edgeR's[16] `cpm()` function with `prior.count = 2` to help stabilize fold-changes of extremely low expression genes. TMM normalization factors after filtering are shown in Fig. S16, and compared to pre-filtering values in Fig. S17.

The overall distribution of logCPM values differed by batch (Fig. S18). The peak on the left of Fig. S18 represents undetected genes, and thus is higher in batches later than November 2020 because those library sizes were smaller.

[16]McCarthy DJ, Chen Y, Smyth GK (2012). "Differential expression analysis of multifactor RNA-Seq experiments with respect to biological variation." Nucleic Acids Research, 40(10), 4288-4297.

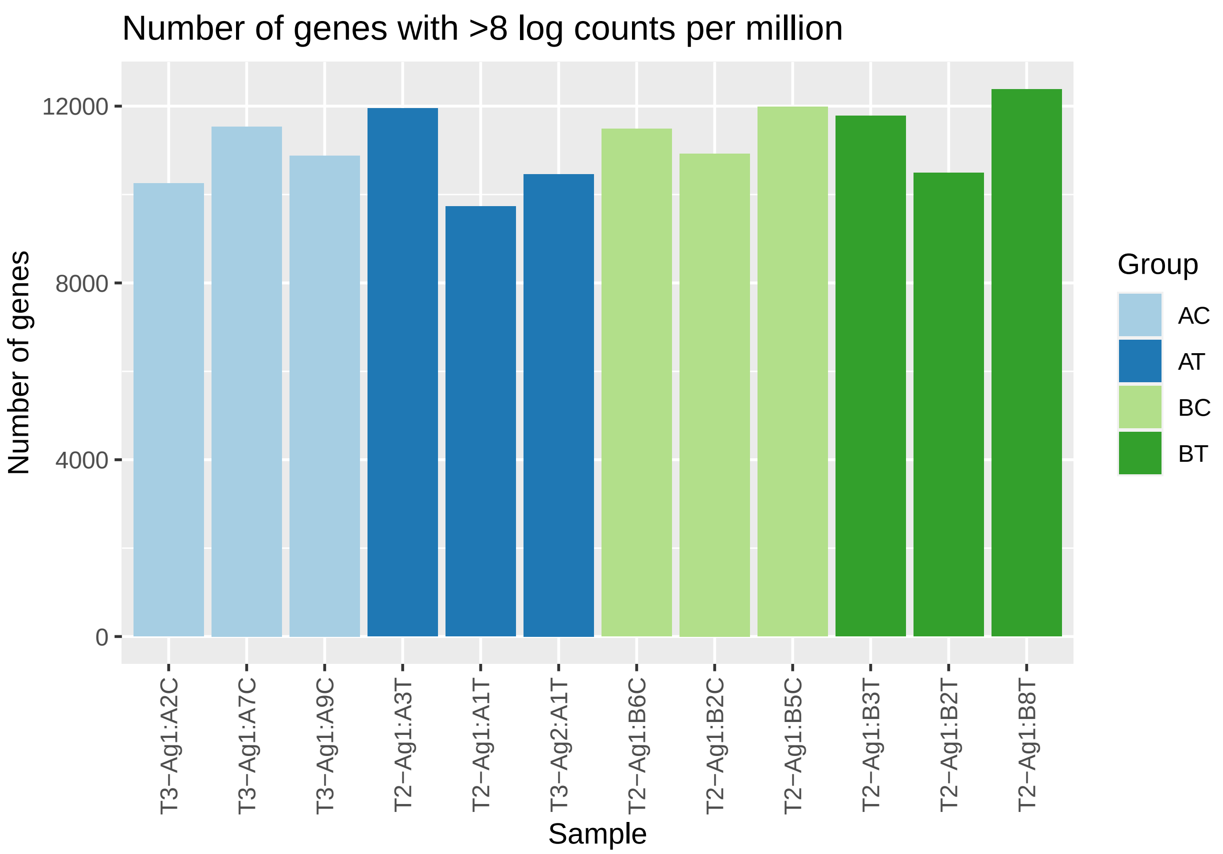

Figure S15: Number of detected genes passing the filtering threshold in each sample.

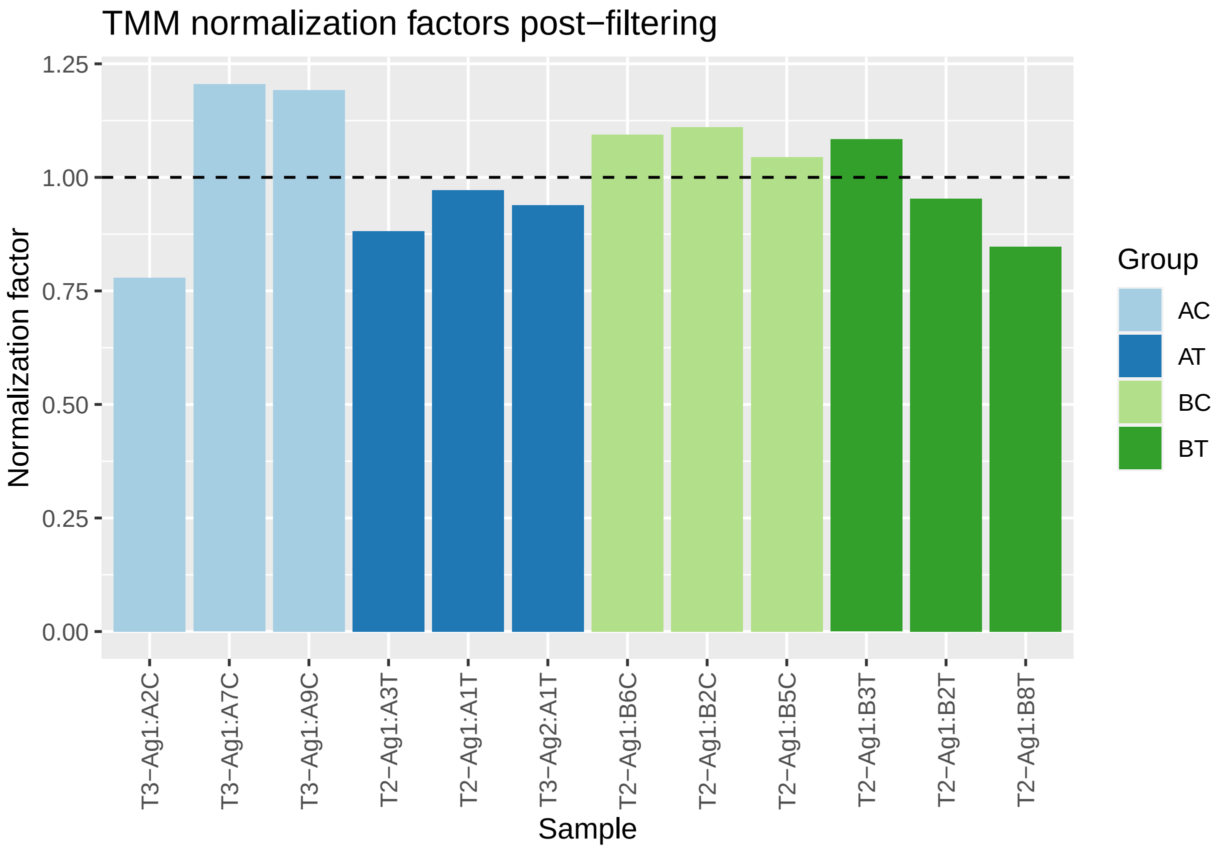

Figure S16: TMM normalization factors post-filtering.

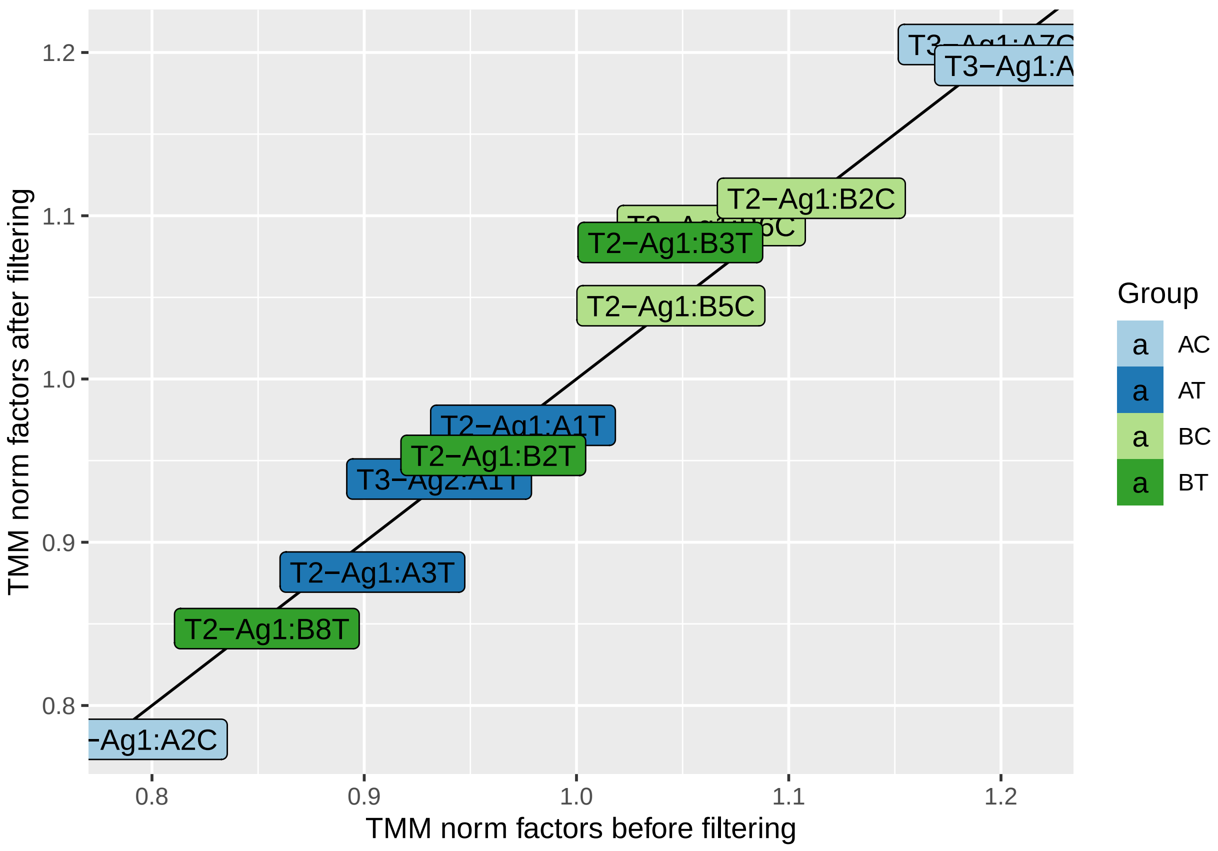

Figure S17: Comparison of TMM normalization factors before and after filtering.

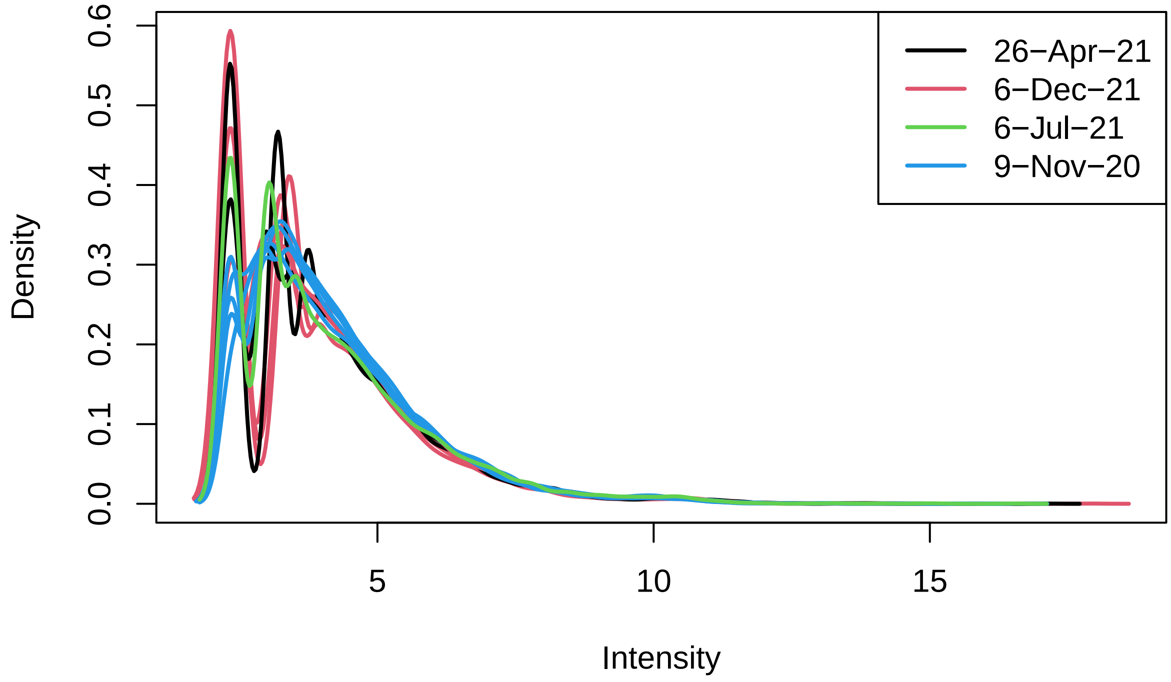

Figure S18: Distribution of logCPM values after filtering.

Sample clustering

Multidimensional scaling in the limma[17] package was used to assess the largest effects on gene composition among samples. The normalized logCPM values of the top most 5,000 variable genes between each pair of samples were chosen to construct the multidimensional scaling plot. The first axis separated the two crosses, while the second axis separated sample T3-Ag1:A2C from the rest and to a lesser extent separated C and T samples (Fig. S19).

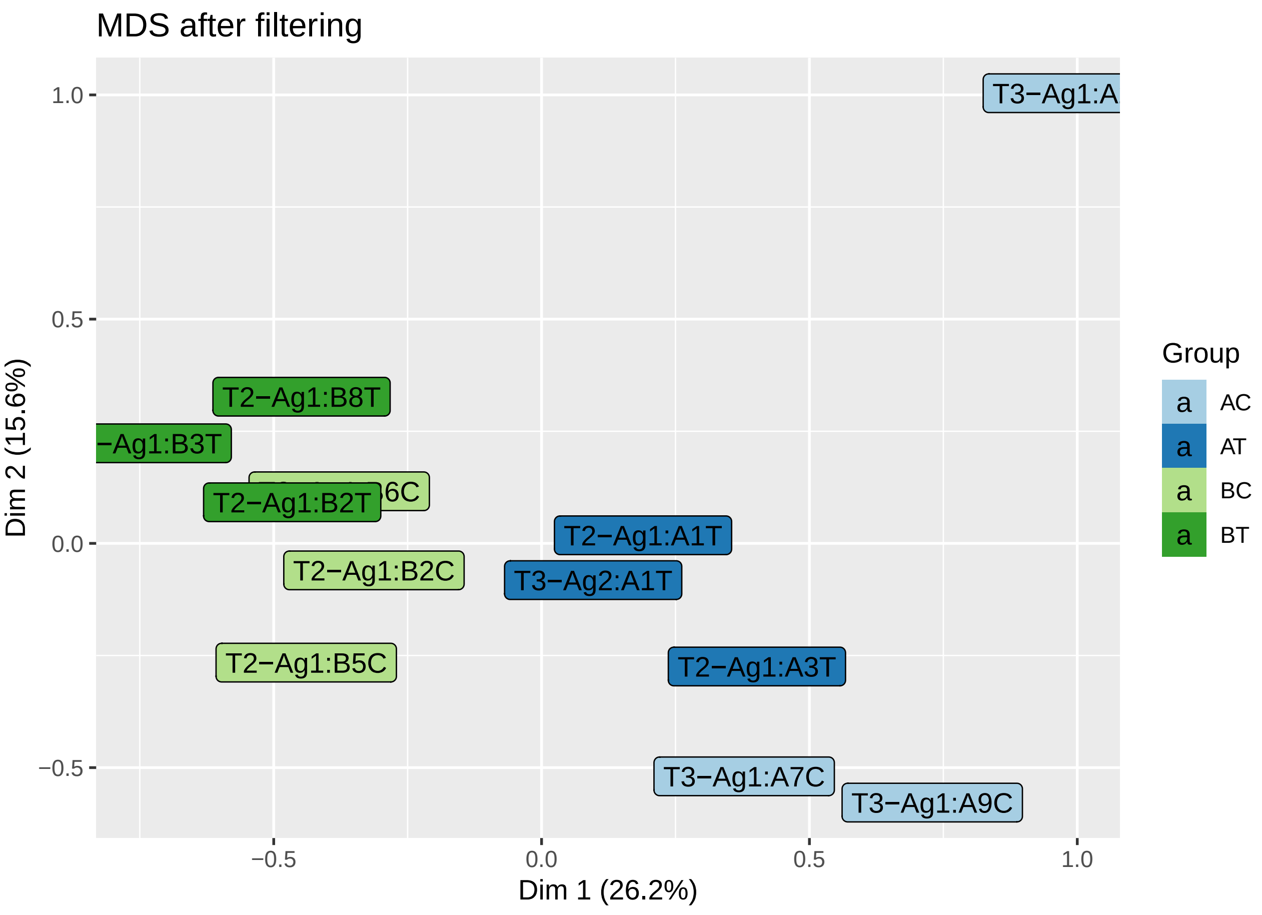

Figure S19: Multidimensional scaling of samples after filtering, using the top 5000 variable genes between each pair of samples.

Differential gene expression

Differential gene expression (DE) analysis was performed using the limma-trend method[18]. Multiple testing correction was done using the False Discovery Rate method[19]. The model run was:

$Y = Group$

where *Group* refers to the AC, AT, BC, and BT groups. The following contrasts were then assessed:

[17] Ritchie, M.E., Phipson, B., Wu, D., Hu, Y., Law, C.W., Shi, W., and Smyth, G.K. (2015). limma powers differential expression analyses for RNA-sequencing and microarray studies. Nucleic Acids Research 43(7), e47.

[18] Chen Y, Lun ATL and Smyth GK. From reads to genes to pathways: differential expression analysis of RNA-Seq experiments using Rsubread and the edgeR quasi-likelihood pipeline [version 2; referees: 5 approved]. F1000Research 2016, 5:1438 (doi: 10.12688/f1000research.8987.2)

[19] Benjamini, Y., & Hochberg, Y. (1995). Controlling the false discovery rate: a practical and powerful approach to multiple testing. Journal of the Royal statistical society: series B (Methodological), 57(1), 289-300.

- **BT - BC**: Differences between T and C bees from the B cross
- **AT - AC**: Differences between T and C bees from the A cross
- **BC - AC**: Differences between B and A crosses, with C as the baseline
- **(BT + AT) - (BC + AC)**: Differences between T and C bees across both crosses
- **(BT + BC) - (AT + AC)**: Differences between crosses, looking at both C and T
- **(BT - BC) - (AT - AC)**: Interaction effect; differential effect of cross on differences between T and C bees

Numbers of significantly up- and down-regulated genes are shown in Table S4.

Table S 4: Number of gene significant for differential expression at FDR < 0.05.

	BT - BC	AT - AC	BC - AC	(BT + AT) - (BC + AC)	(BT + BC) - (AT + AC)	(BT - BC) - (AT - AC)
**Down**	**0**	**0**	**133**	**0**	**306**	**0**
NotSig	14461	14461	13922	14462	13465	14462
**Up**	**1**	**1**	**407**	**0**	**691**	**0**

Heatmaps for differential gene expression

Across all of the experimental groups contrasts, a one-way ANOVA was run in limma, identifying 619 significant genes. These genes were then plotted in a heatmap to summarize gross changes in gene expression across groups. Most of the significant genes displayed consistent expression differences between cross A and cross B (Fig. S20).

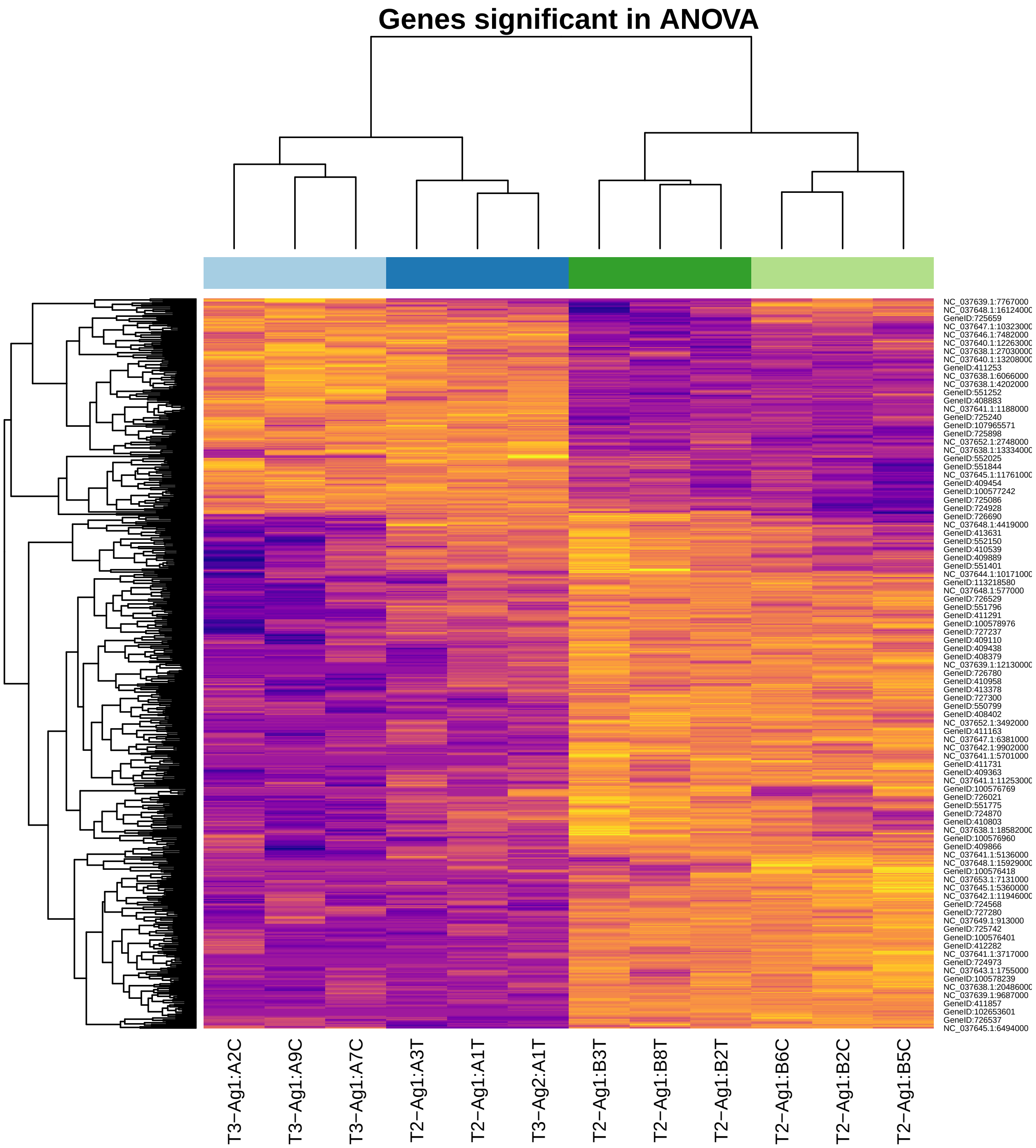

Figure S20: Heatmap of 582 genes with FDR < 0.05 in one-way anova for effect of experimental group. Unadjusted expression values are shown.

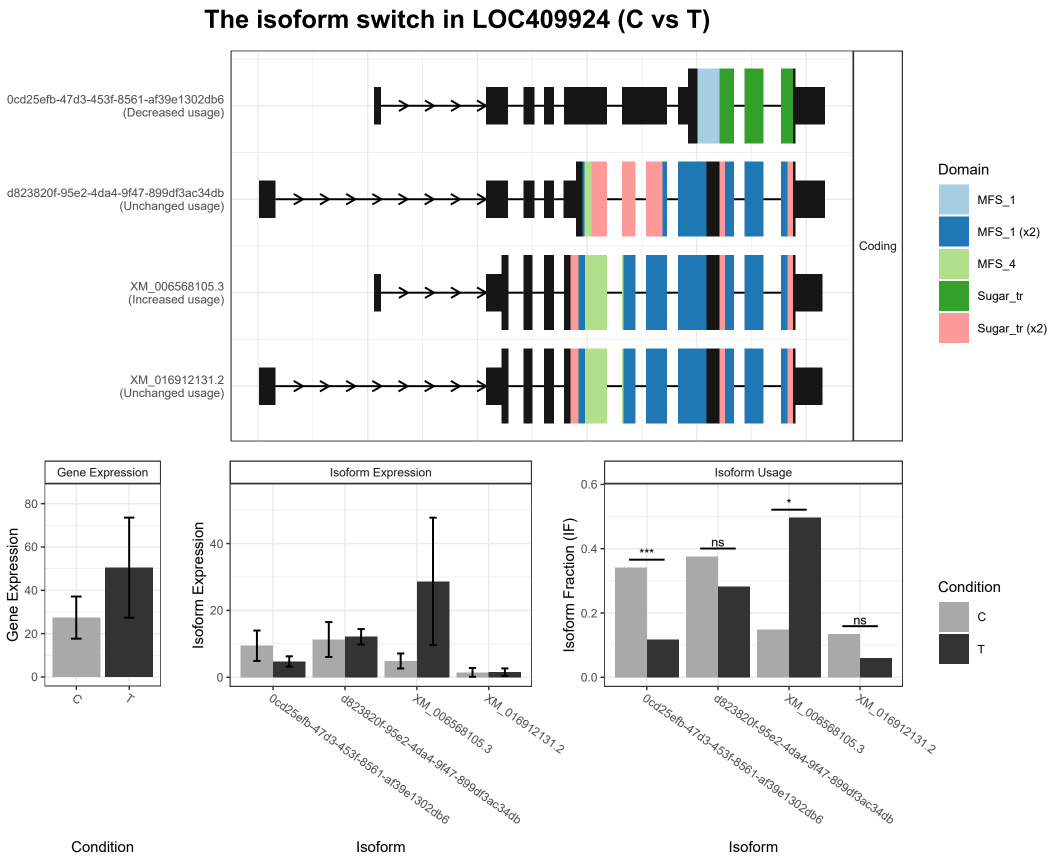

Figure S21: Isoform switch in LOC409924 (C vs T)

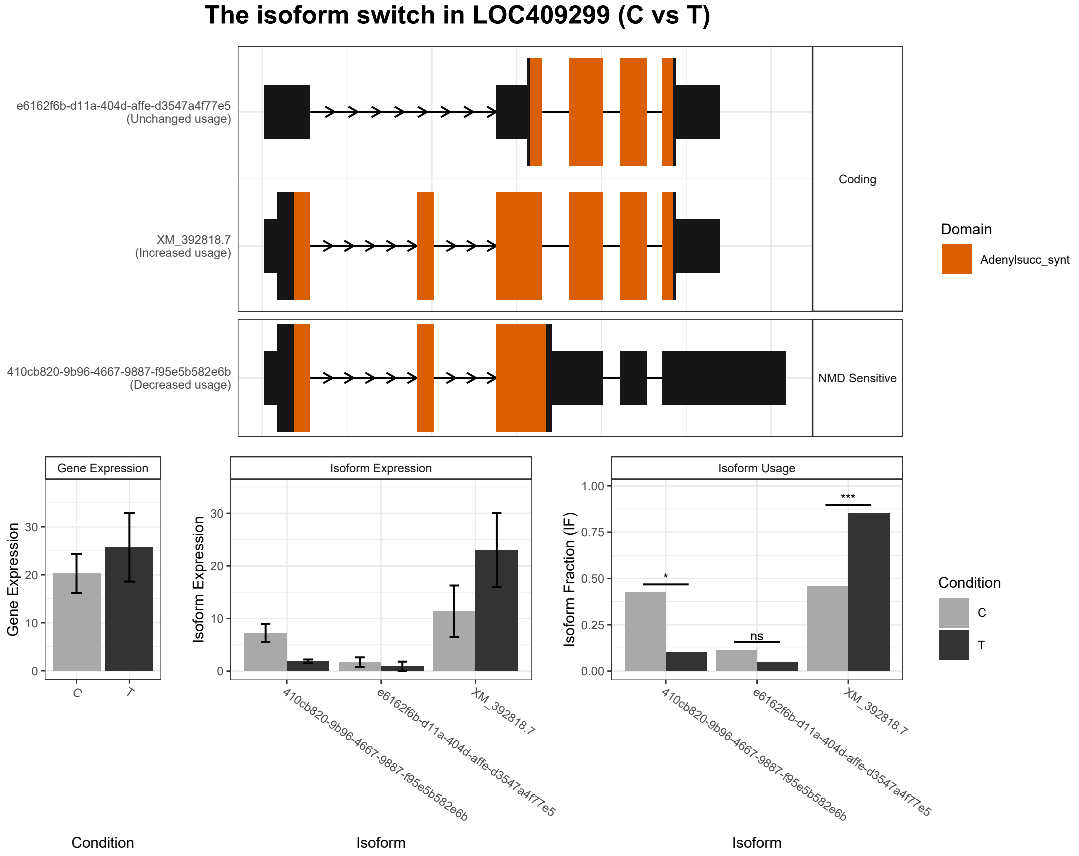

Figure S22: Isoform switch in LOC409299 (C vs T)

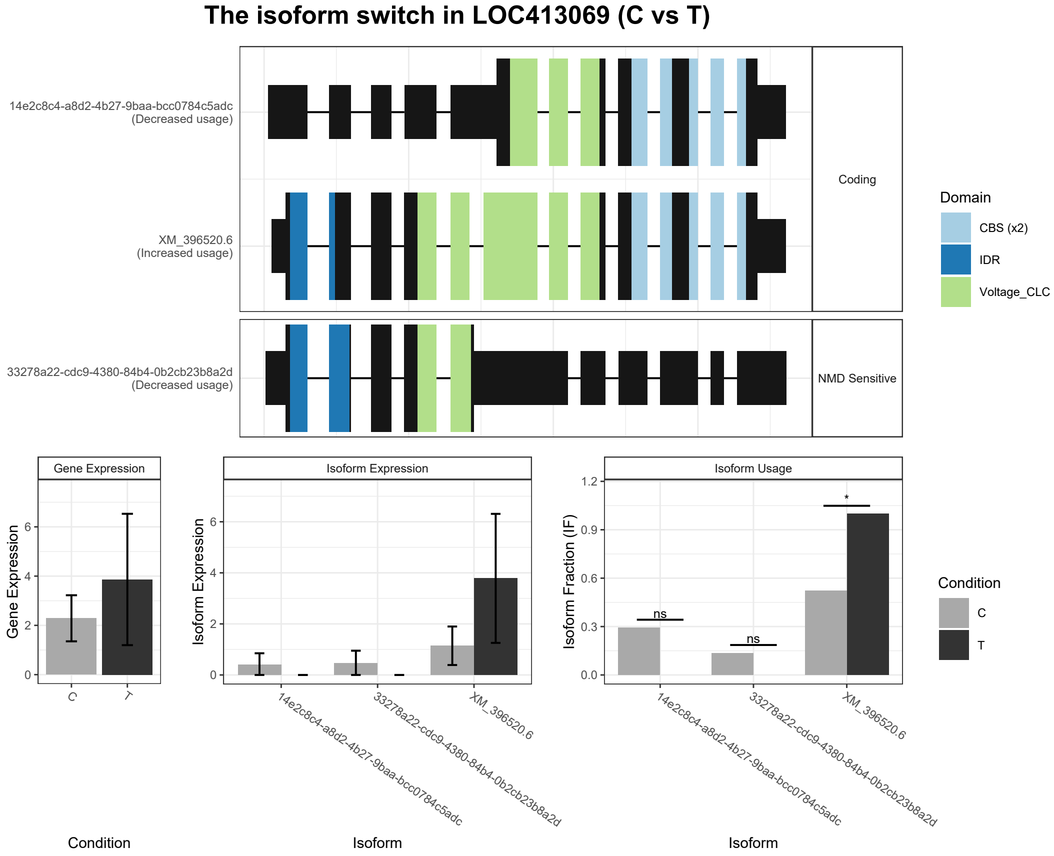

Figure S23: Isoform switch in LOC413069 (C vs T)

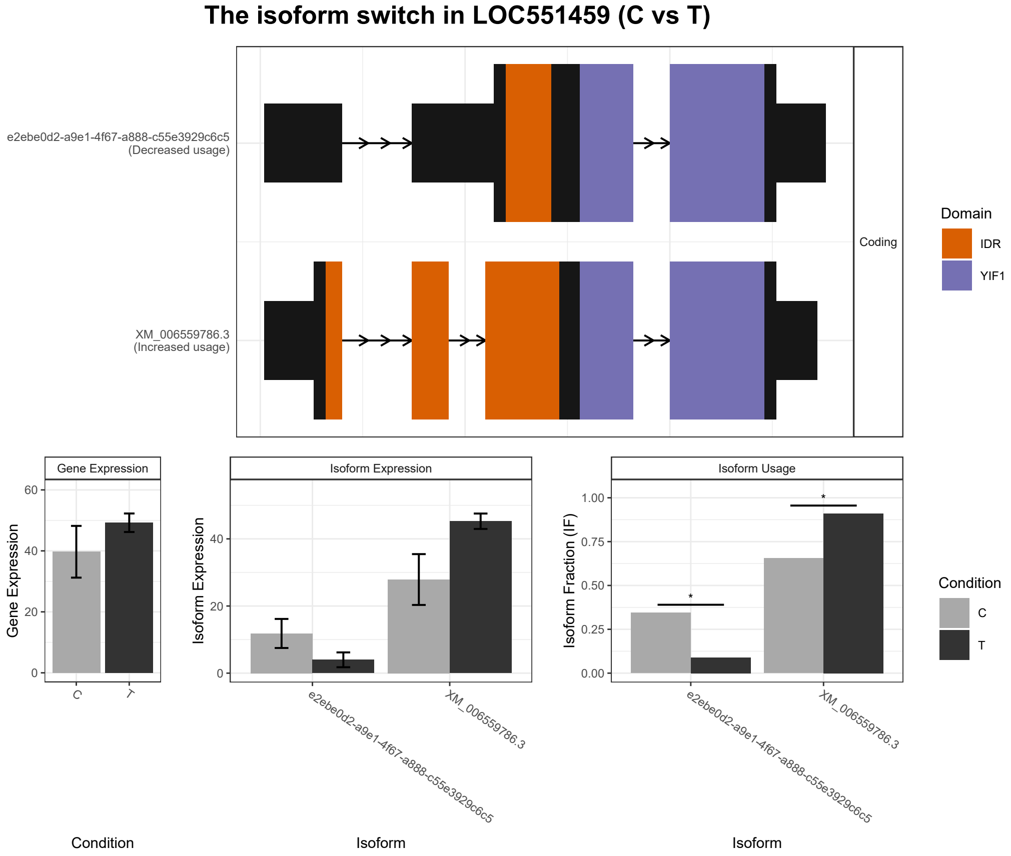

Figure S24: Isoform switch in LOC551459 (C vs T)

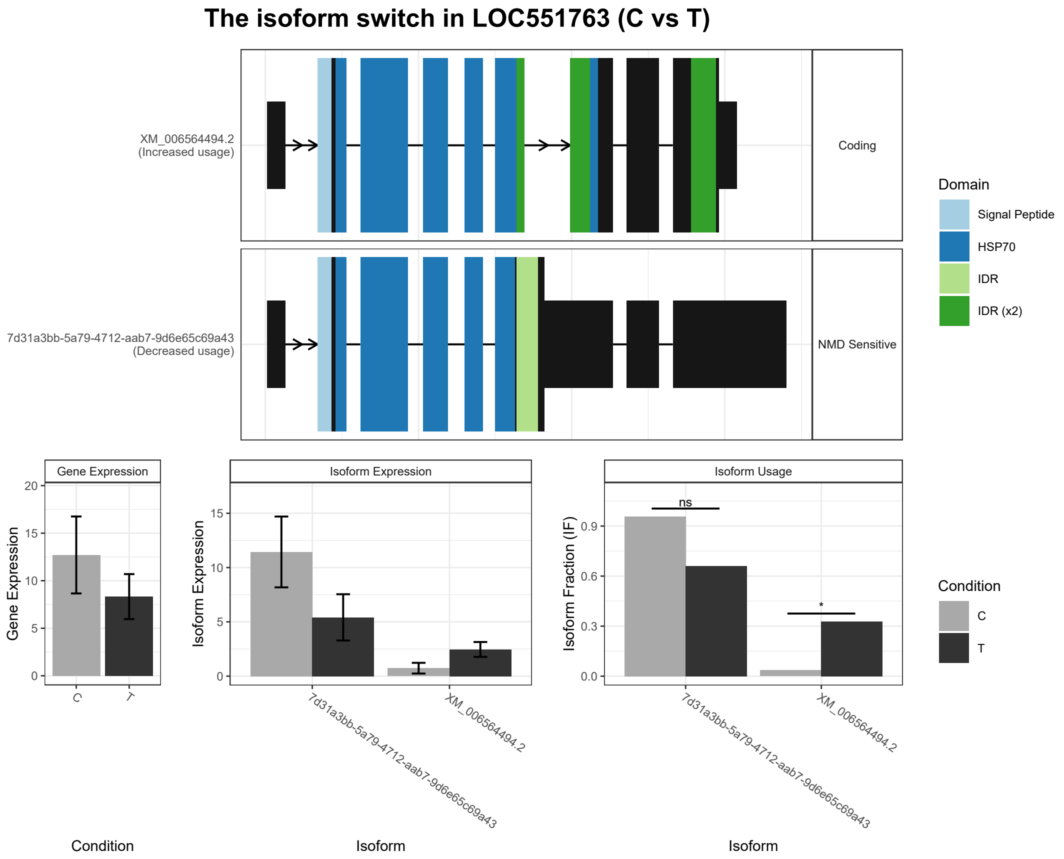

Figure S25: Isoform switch in LOC551763 (C vs T)

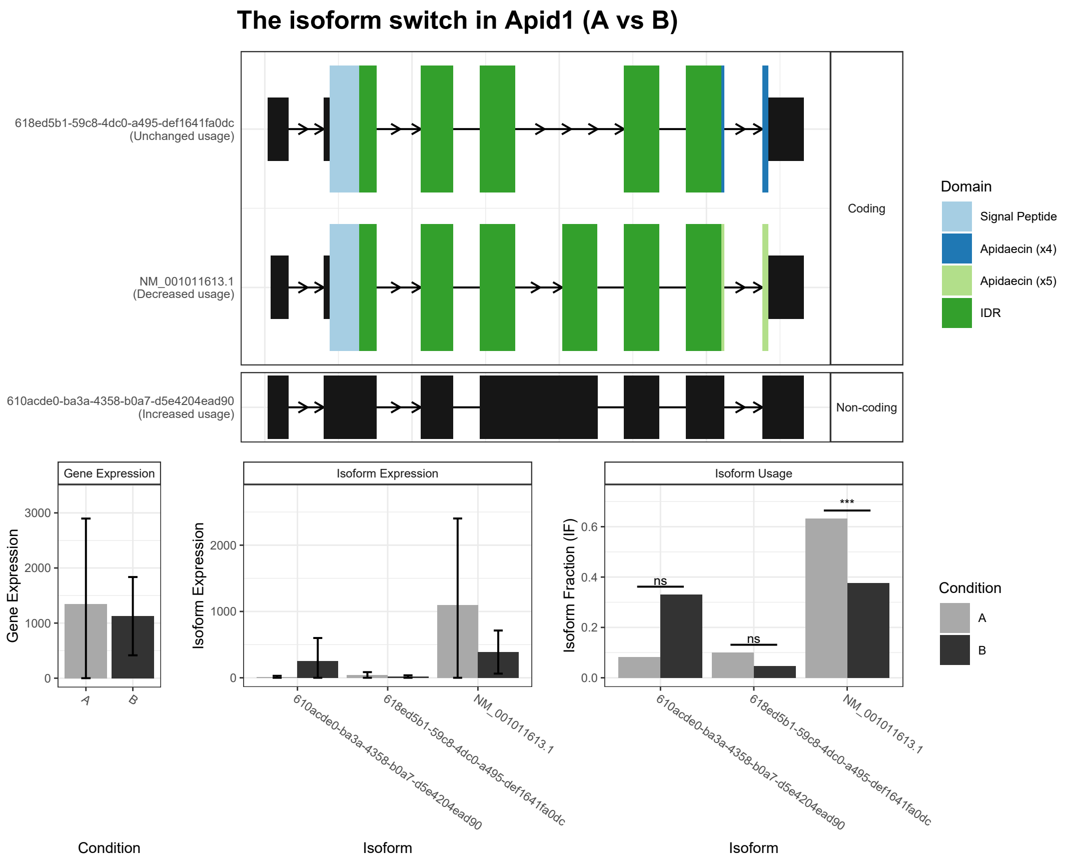

Figure S26: Isoform switch in Apid1 (A vs B)

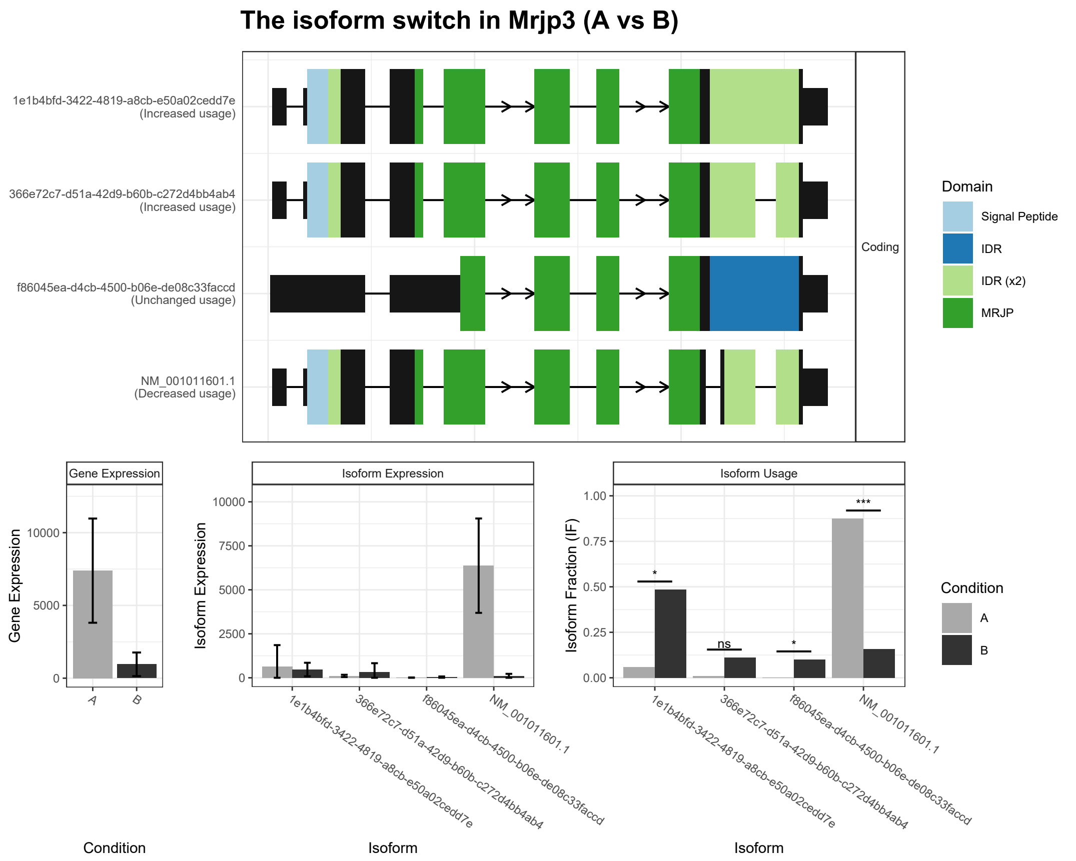

Figure S27: Isoform switch in Mrjp3 (A vs B)

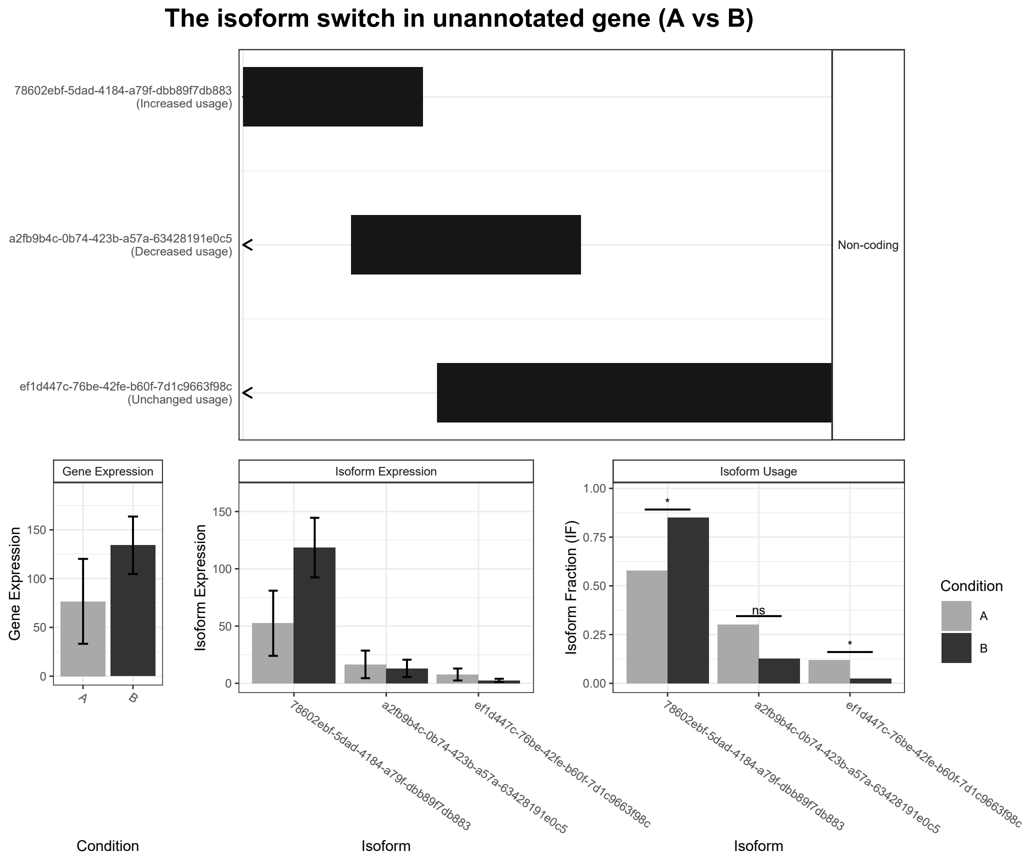

Figure S28: Isoform switch in unannotated gene (A vs B)

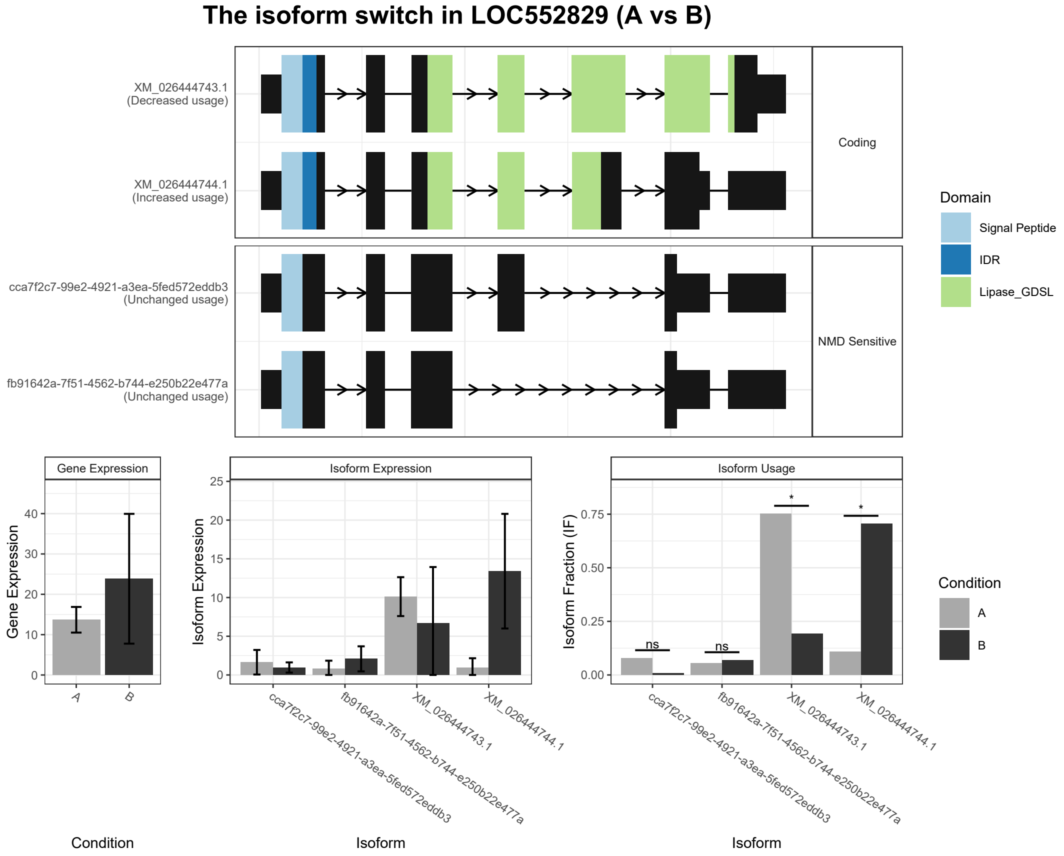

Figure S29: Isoform switch in LOC552829 (A vs B)

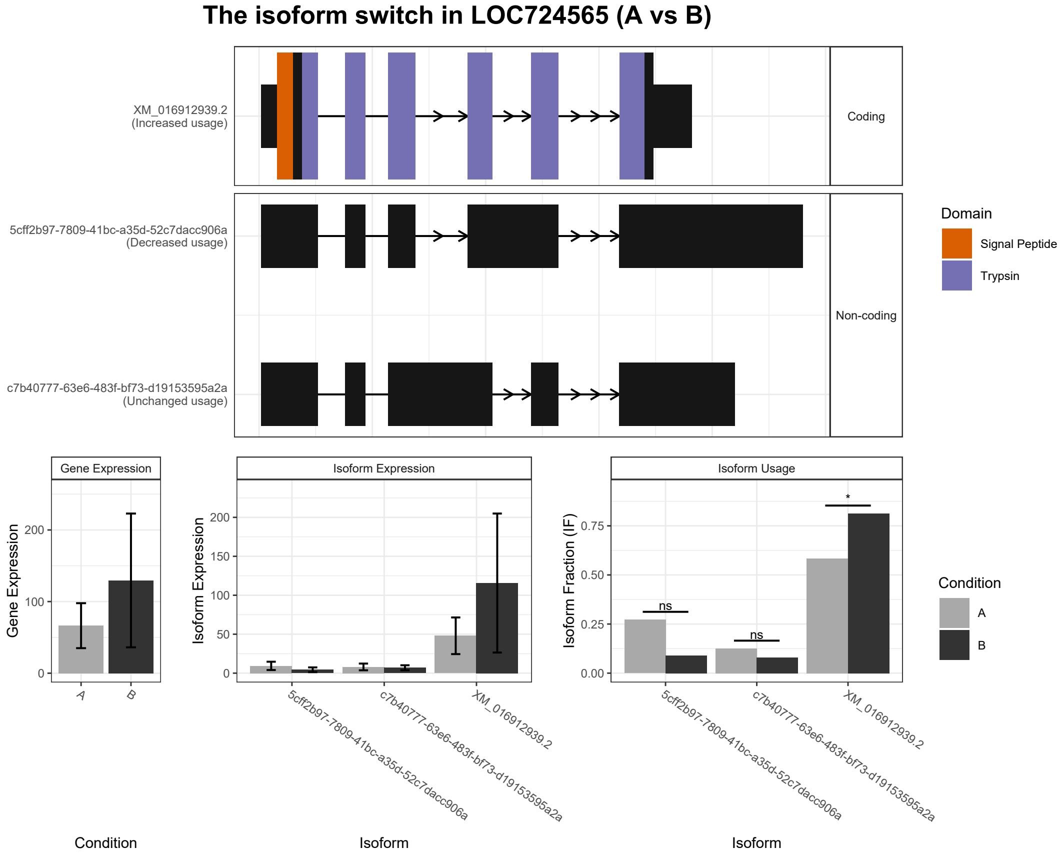

Figure S30: Isoform switch in LOC724565 (A vs B)

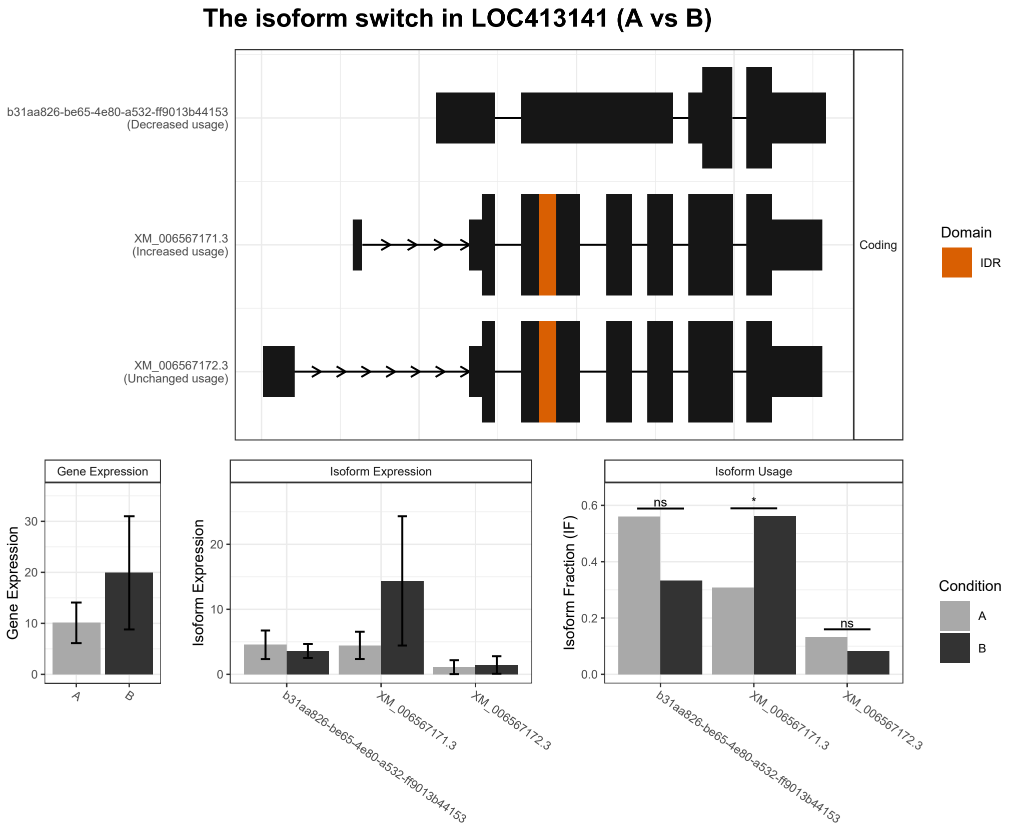

Figure S31: Isoform switch in LOC413141 (A vs B)

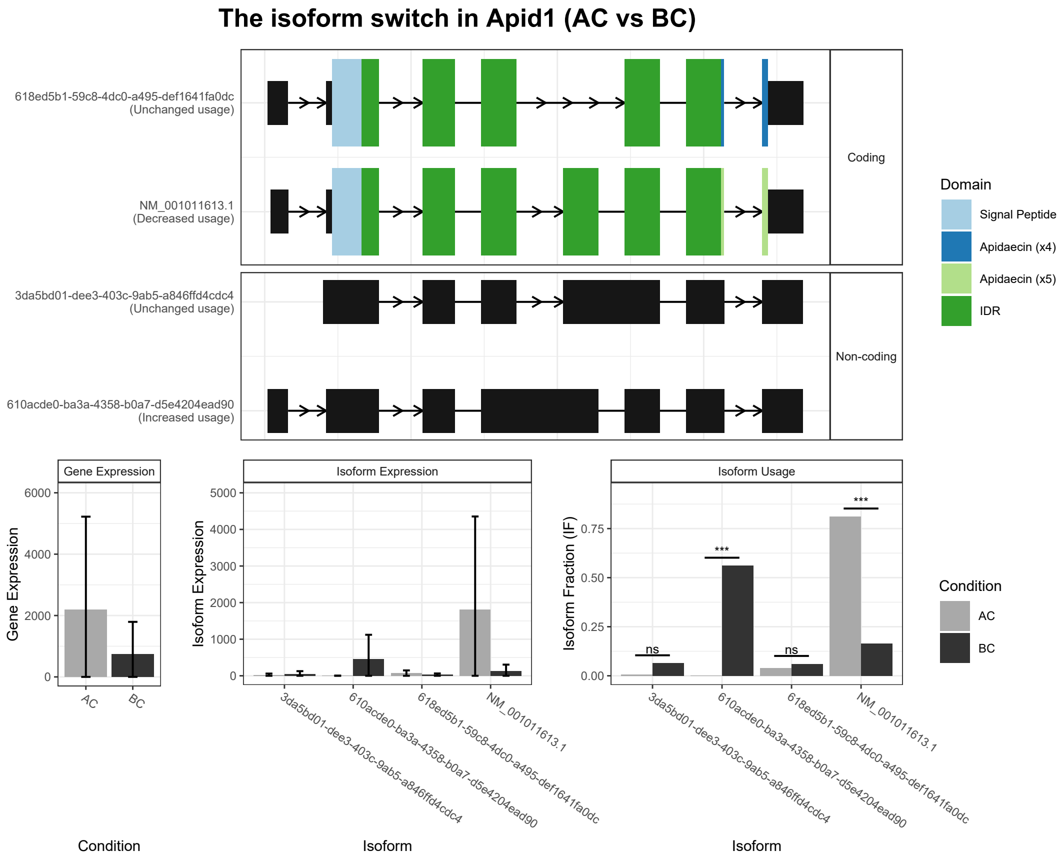

Figure S32: Isoform switch in Apid1 (AC vs BC)

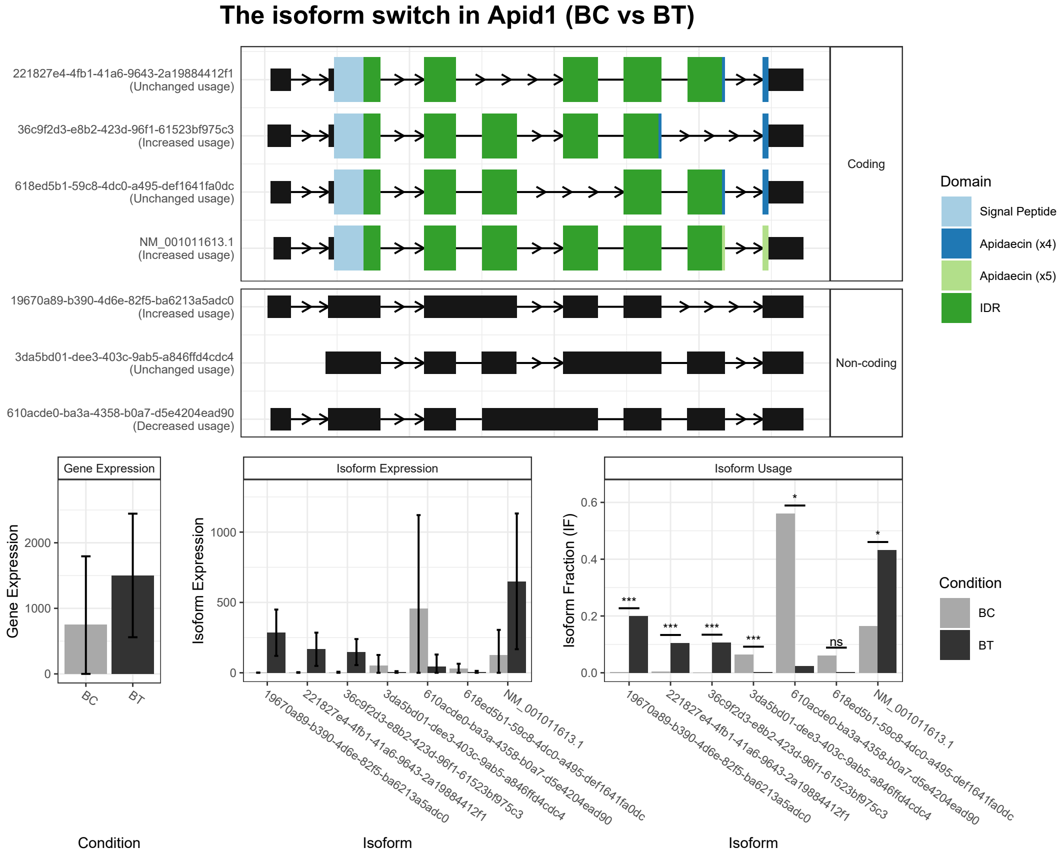

Figure S33: Isoform switch in Apid1 (BC vs BT)

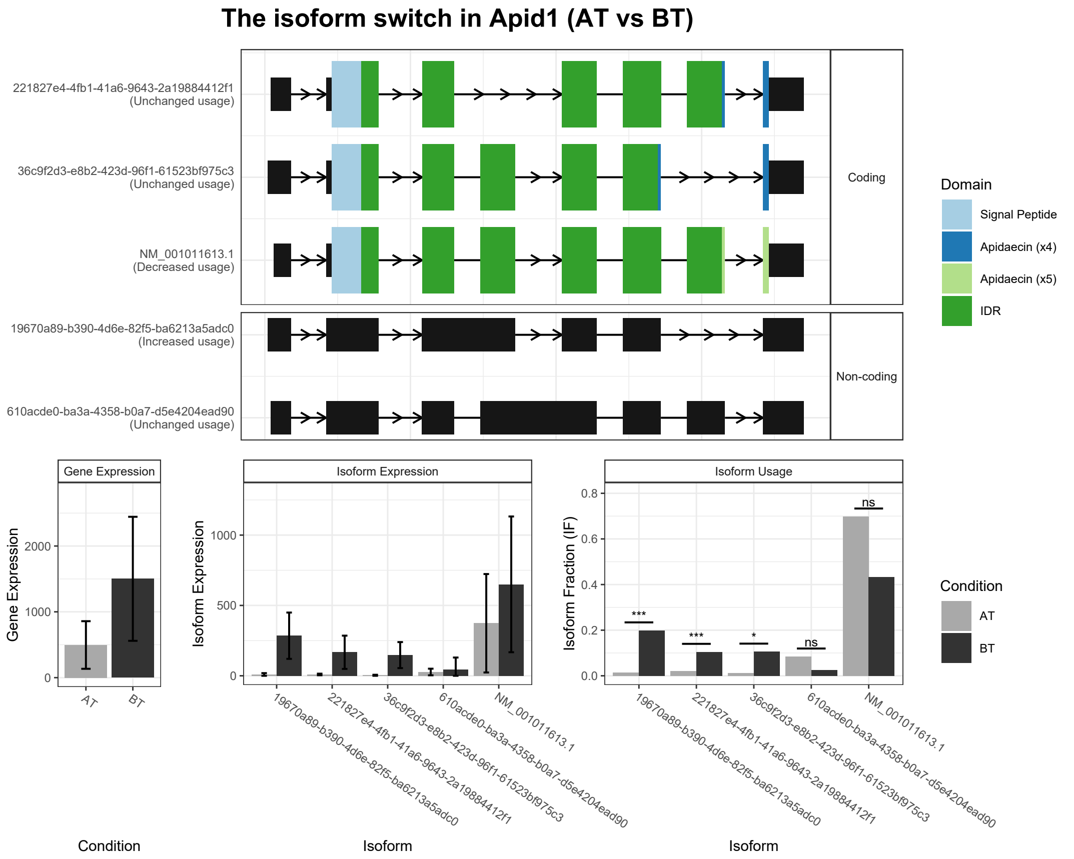

Figure S34: Isoform switch in Apid1 (AT vs BT)

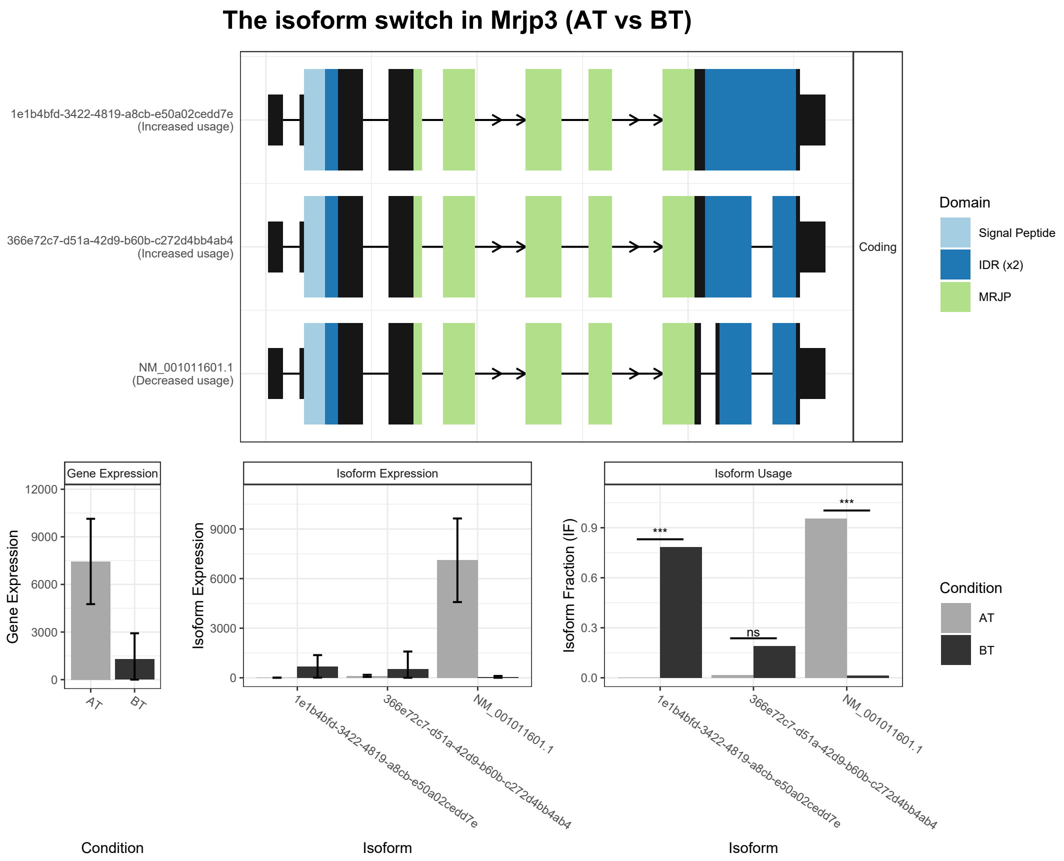

Figure S35: Isoform switch in Mrjp3 (AT vs BT)

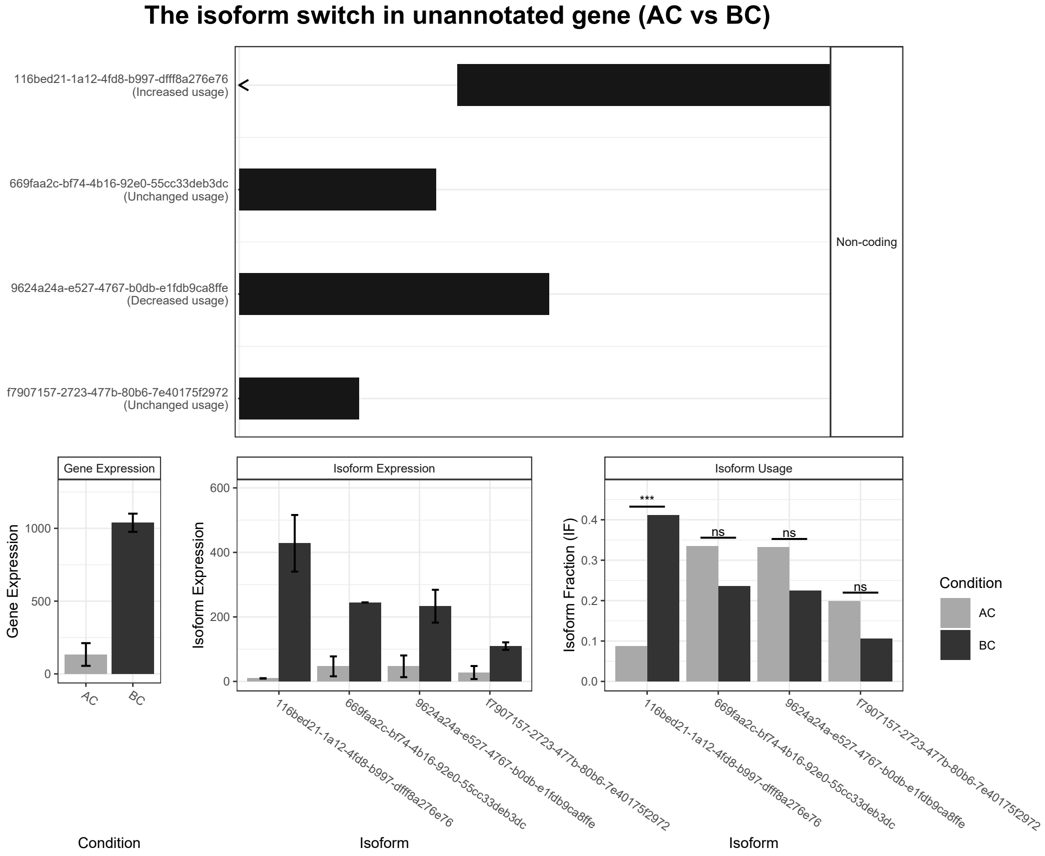

Figure S36: Isoform switch in unannotated gene (AC vs BC)

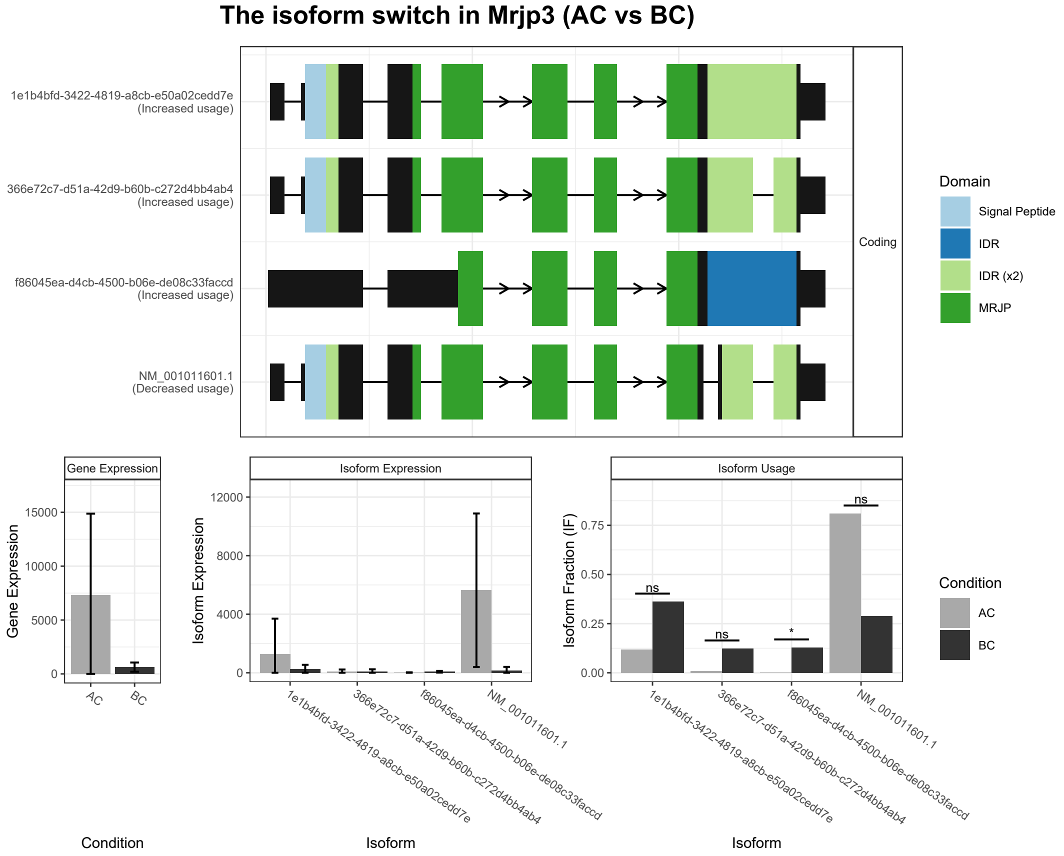

Figure S37: Isoform switch in Mrjp3 (AC vs BC)

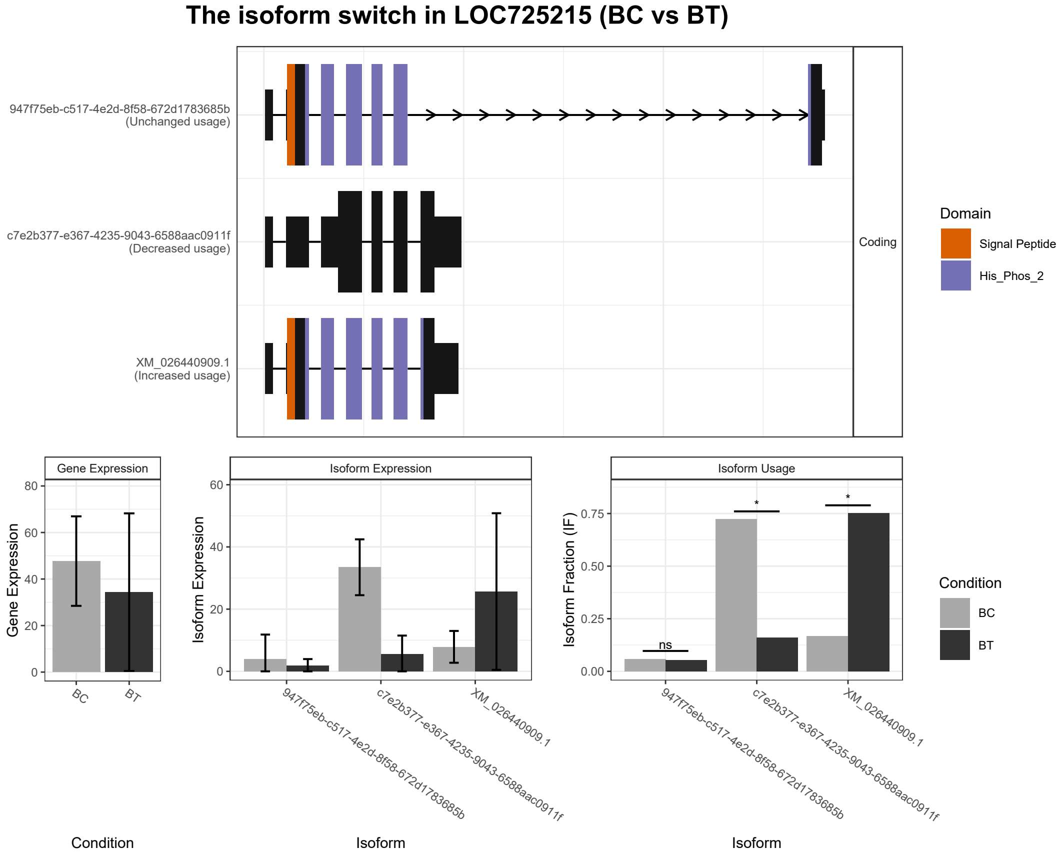

Figure S38: Isoform switch in LOC725215 (BC vs BT)

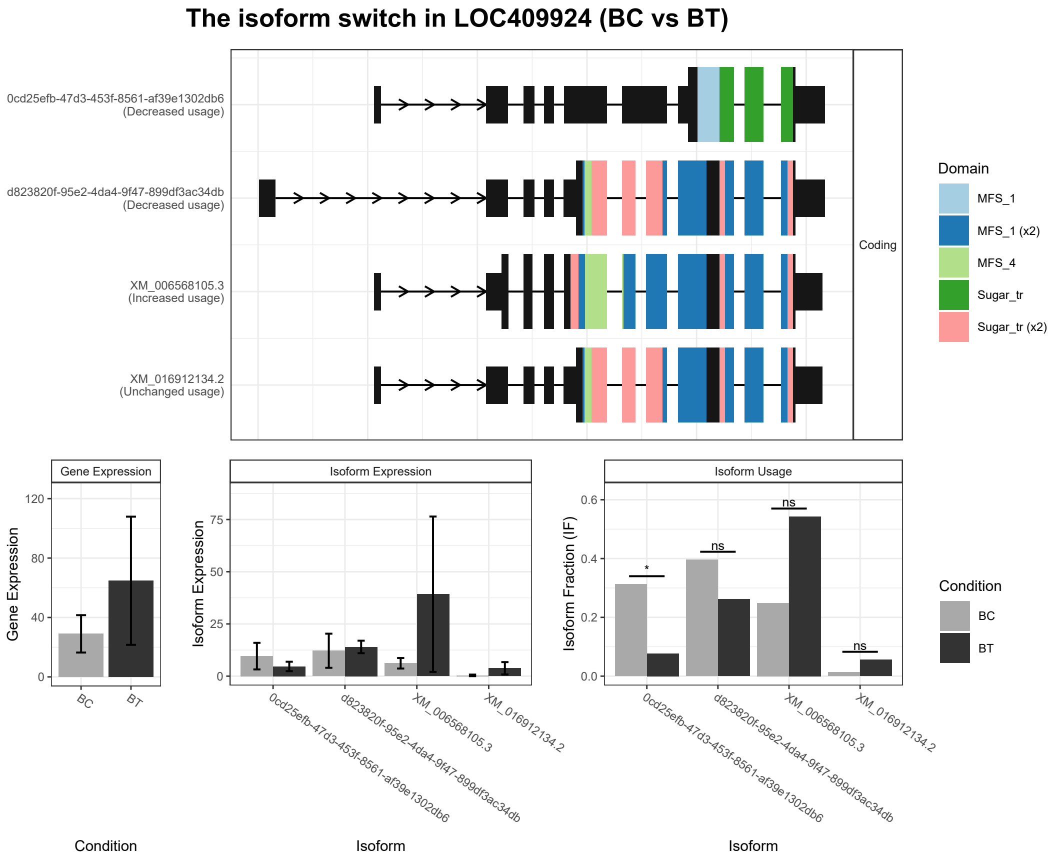

Figure S39: Isoform switch in LOC409924 (BC vs BT)

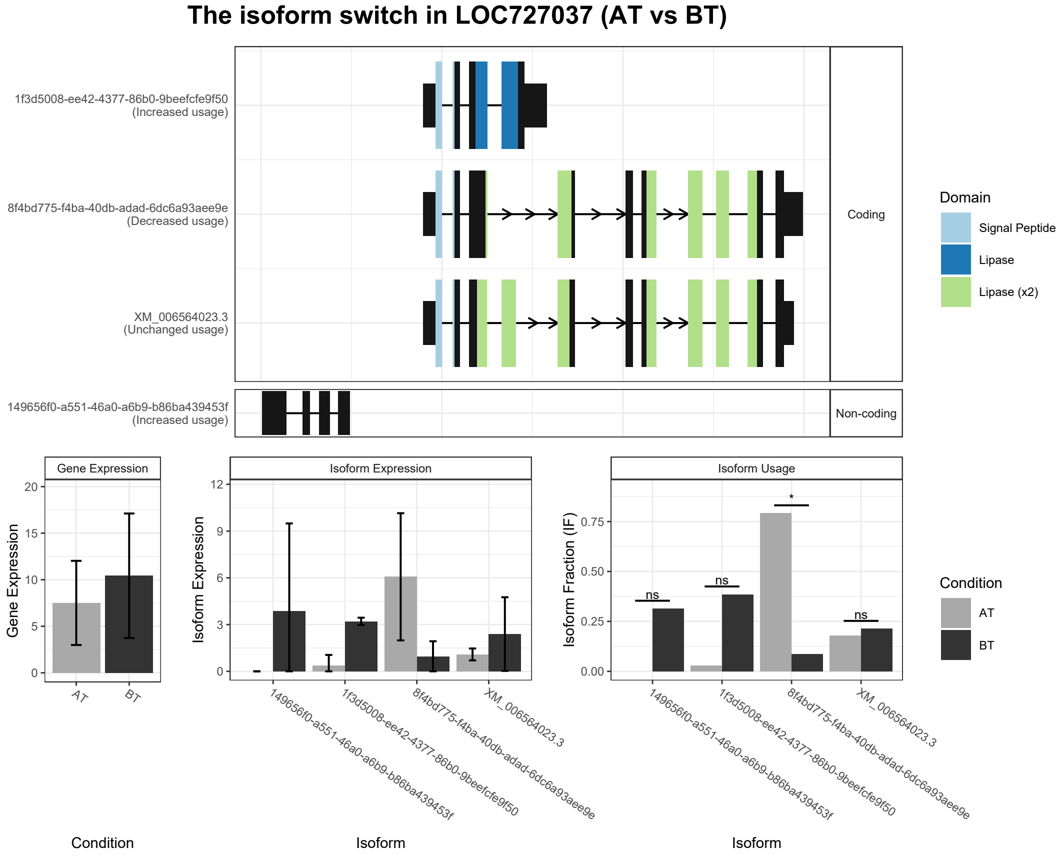

Figure S40: Isoform switch in LOC727037 (AT vs BT)

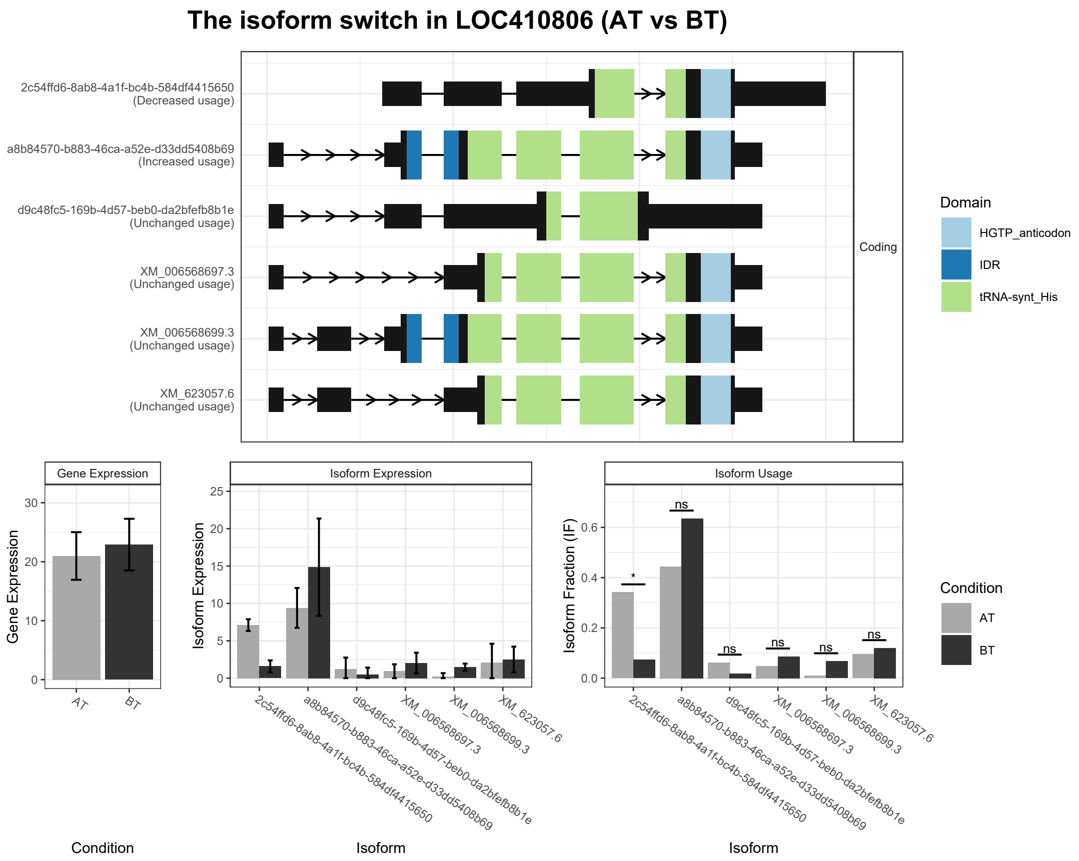

Figure S41: Isoform switch in LOC410806 (AT vs BT)

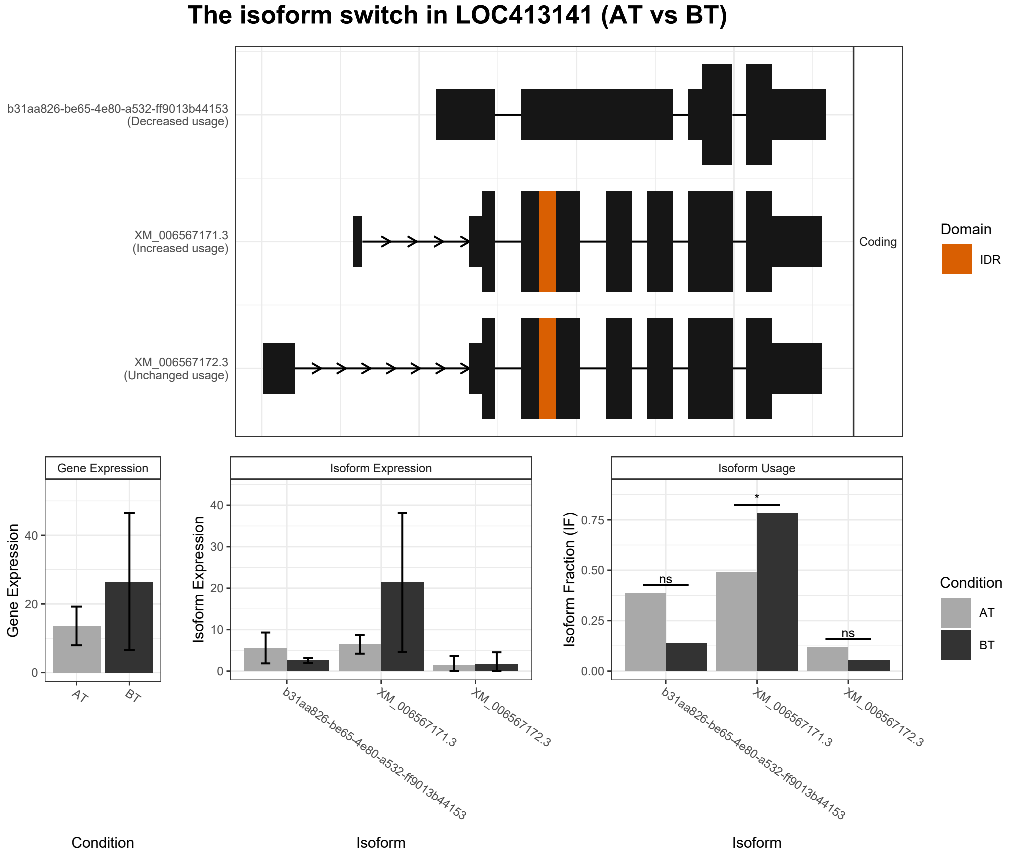

Figure S42: Isoform switch in LOC413141 (AT vs BT)

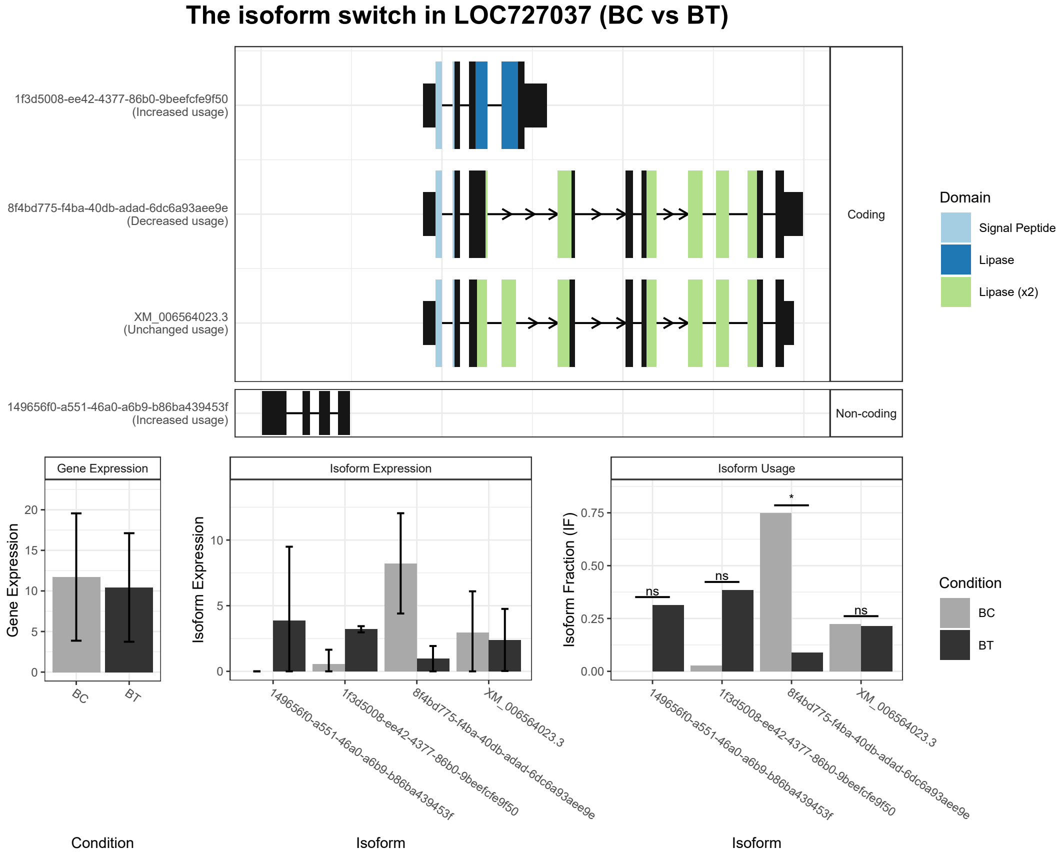

Figure S43: Isoform switch in LOC727037 (BC vs BT)

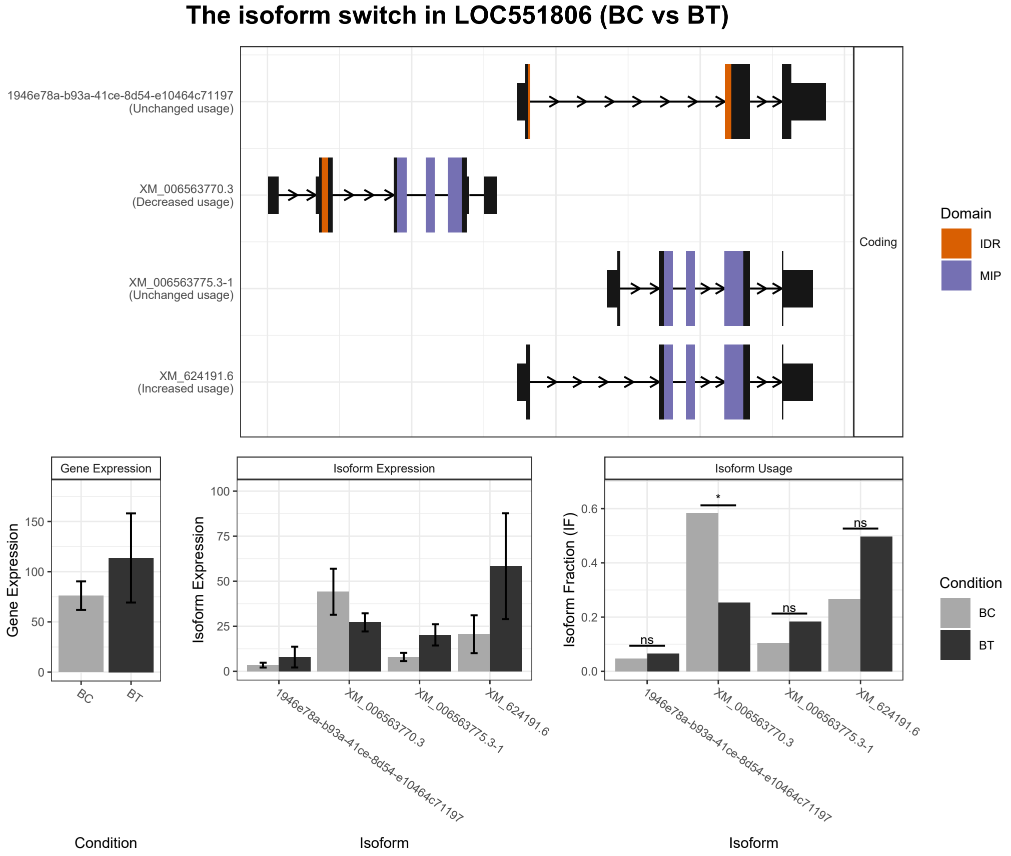

Figure S44: Isoform switch in LOC551806 (BC vs BT)
